# Effects of different interventions based on virtual reality on cognitive and motor function in elderly people with mild cognitive impairment or dementia: a systematic review and network meta-analysis

**DOI:** 10.3389/fnagi.2026.1888654

**Published:** 2026-08-10

**Authors:** Jingsen Liu, Kaixuan Sui, Luhao Liu, Jia Song, Lijuan Yang, Minmin Leng

**Affiliations:** 1 Department of Nursing, Shandong Provincial Hospital Affiliated to Shandong First Medical University, Jinan, Shandong, China; 2 School of Nursing, Shandong First Medical University, Jinan, China

**Keywords:** virtual reality interventions, mild cognitive impairment, dementia, cognitive function, motor function, network meta-analysis

## Abstract

Virtual reality (VR)-based interventions are recognized as promising non-pharmacological therapies for enhancing cognitive and motor function in elderly patients with cognitive impairment. However, the comparative effectiveness of different VR-based interventions remains to be established. To address this research gap, a frequentist framework was utilized to conduct a network meta-analysis that integrated both direct and indirect comparative evidence. A comprehensive electronic literature search was conducted in six English databases and three Chinese databases to identify relevant randomized controlled trials (RCTs) published up to October 2025. A random effects model was selected for traditional meta-analysis to directly examine the efficacy of different non-pharmacological interventions. A consistency model was chosen to perform a network meta-analysis to evaluate the relative effects and rank probabilities of other non-pharmacological interventions. A total of 42 randomized controlled trials (RCTs) were included, encompassing four distinct non-pharmacological interventions. Risk of bias was assessed with the Cochrane Risk of Bias 2 tool, and certainty of evidence with CINeMA (Confidence in NMA). Preliminary evidence suggests that virtual reality sport training (VR-SPT) may positively affect cognitive outcomes in patients with cognitive impairment, particularly those with mild cognitive impairment (MCI). However, the evidence supporting the executive function benefits is extremely limited. Specifically, the potential advantages of VR-SPT for executive function rest on only two studies, and the confidence intervals reported were very wide, rendering these findings highly uncertain. Concurrently, the available evidence suggests that VR comprehensive cognitive game combined with sport training (VR-CCG+SPT) may offer a potential advantage in improving motor function in patients with cognitive impairment. Furthermore, in patients with dementia, VR-CCG+SPT also demonstrated more favorable effects on global cognitive function. This network meta-analysis provides a comprehensive comparison of four different virtual reality (VR) interventions on cognitive and motor function in patients with cognitive impairment, offering preliminary evidence for clinical practice and future research. The selection of VR interventions should be guided by the patient’s living environment, personal preferences, and the aforementioned preliminary findings, to delay cognitive and motor function decline. However, due to limitations such as a relatively weak evidence base for key comparisons, overly wide confidence intervals for some results, and the use of overlapping MoCA domains in different outcome networks, the results of this study should be interpreted with caution, and the above conclusions require further research for validation. Future research should prioritize conducting well-designed, multi-arm randomized controlled trials to provide more robust evidence concerning the relative efficacy of various VR interventions.

**Systematic review registration:**

https://www.crd.york.ac.uk/PROSPERO/view/CRD420251034623, identifier CRD420251034623.

## 1 Introduction

The accelerating aging of the global population has resulted in a substantial increase in the number of older adults with cognitive impairments. Cognitive impairments are clinically classified as dementia or mild cognitive impairment (MCI). Dementia is defined as a clinical syndrome characterized by a deterioration in various cognitive functions—including memory, language, attention, visuospatial ability, and frontal executive function—that impairs daily living activities (Tartaglia et al., 2011). Additionally, MCI is recognized as a prodromal stage of dementia, with cognitive performance being situated between normal aging and dementia (Kelley and Petersen, 2007). According to a 2025 World Health Organization (WHO) report dementia affected 57 million individuals worldwide in 2021 (World Health Organization [WHO], 2025), with projections indicating an increase to 152 million by 2050 (GBD 2019 Dementia Forecasting Collaborators, 2022). Similarly, the global prevalence of mild cognitive impairment in geriatric population was found 23.7% (Salari et al., 2025), thereby affecting approximately 50 million individuals worldwide in 2020; moreover, this prevalence is estimated to exceed 150 million by 2050 (Livingston et al., 2020; Nichols et al., 2022). Cognitive decline manifests as memory loss, executive dysfunction, attentional deficits, and reduced capacity for daily living activities (Arevalo-Rodriguez et al., 2021; Amrapala et al., 2023), impaired ADL and IADL are found in people with dementia, while MCI patients are necessarily still independent in these activities (Petersen, 2016), all of which contribute to diminished quality of life for patients and caregivers while imposing a significant burden on health care systems.

Health management for older adults with cognitive impairments includes both pharmacological and non-pharmacological interventions (Hugo and Ganguli, 2014; Orgeta et al., 2020). Pharmacotherapy is currently limited by limited drug options, significant side effects, and prognostic uncertainty (Dale et al., 2006; Odawara, 2012). Traditional non-pharmacological approaches, include self-guided training, dietary management, physical exercise, and social engagement (Eshkoor et al., 2015). Its effectiveness is often affected by the insufficient standardization and personalization of the intervention process, as well as the lack of sufficient interest and immersion in the training content (Lasaponara et al., 2021). With recent technological advancements, virtual reality (VR) has emerged as a promising non-pharmacological intervention (Salim and Taylor, 2026). VR enables the construction of highly simulated three-dimensional environments via multisensory integration (including visual, auditory, and tactile sensations). These immersive scenarios have been demonstrated to increase attention and motivation during training sessions (Papaioannou et al., 2022). Studies suggest that VR training can strengthen functional connectivity within the frontal-parietal cortical network, which aligns with established associations between neuronal plasticity and cognitive improvement (W hlin et al., 2019). Moreover, patient-specific parameter adjustments (including task complexity and feedback latency) allow for customized difficulty levels to be established, thereby improving compliance (Gómez-Cáceres et al., 2023; Goumopoulos et al., 2023); additionally, complex motor tasks (such as obstacle avoidance and balance training) can be safely simulated while mitigating real-world safety risks (Padala et al., 2012; Ip et al., 2025). Furthermore, immediate behavioral data collection (including reaction times and motor trajectories) facilitates corrective feedback to optimize learning reinforcement (Amjad et al., 2019; Arshad et al., 2023).

Recent randomized controlled trials (RCTs) and traditional meta-analyses have substantiated the beneficial effects of diverse VR-based non-pharmacological interventions on cognitive and motor functions. For example, compared with control games, comprehensive VR games have been shown to significantly enhance global cognition and executive functions (Kang et al., 2021; Arshad et al., 2023; Goumopoulos et al., 2023). Similarly, studies on combined VR Comprehensive Cognitive Game with Sport Training have reported significant improvements in cognition, gait speed, and balance in patients with mild cognitive impairment (MCI) (Hagovská and Olekszyová, 2016; Schwenk et al., 2016; Ip et al., 2025). Furthermore, research on VR sport training has indicated that it can significantly improve overall motor function, executive function, gait speed, and balance in patients with MCI and Alzheimer’s disease (AD) (Padala et al., 2012; Hsieh et al., 2018). The specific VR modalities evaluated in the literature include VR-based cognitive training (Moulaei et al., 2024; Yu et al., 2024; Yang et al., 2025), VR-assisted motor rehabilitation (Zhao et al., 2020; Ren et al., 2024), combined VR comprehensive cognitive game with sport training (Delbroek et al., 2017; Choi and Lee, 2019; Buele et al., 2024) and VR reminiscence therapy (Tominari et al., 2021). Consequently, VR-based interventions are deemed to be viable therapeutic options for enhancing health outcomes in cognitively impaired older adults. However, direct comparative evidence among distinct VR approaches remains unavailable, and the optimal VR intervention for mitigating cognitive or motor decline in MCI/dementia patients has yet to be established. This evidence gap significantly impedes clinical decision-making regarding the most effective VR-based therapeutic selection.

Network meta-analysis (NMA) is recognized as a novel statistical methodology that integrates direct and indirect evidence to simultaneously quantify treatment effects (Salanti et al., 2008). This approach enables indirect comparisons of ≥ 2 interventions within a unified framework while also estimating treatment rankings and relative efficacy probabilities for each intervention (Ades et al., 2006). Consequently, the RCT data were analyzed in this study via NMA to evaluate the comparative efficacy of different VR-based interventions in preventing cognitive, executive, and motor decline in patients with MCI or dementia, providing preliminary evidence to inform clinical decision-making.

## 2 Methods

This NMA was conducted in accordance with the Preferred Reporting Items for Systematic Reviews and Meta-Analyses guidelines extension statement for Network Meta-Analysis (PRISMA-NMA) (Hutton et al., 2015) and was registered with PROSPERO (CRD420251034623).

### 2.1 Search strategy

A comprehensive electronic literature search was conducted by two independent investigators across six English databases (PubMed, Web of Science, Embase, CINAHL, Cochrane Library and IEEE Xplore) and three Chinese databases (CNKI, Wanfang and VIP) to identify relevant studies published through October 2025. The references of the systematic reviews and meta-analyses were screened to ensure comprehensive coverage. The search strategies were customized for each database by using Boolean combinations of Medical Subject Headings (MeSH) and free-text terms for the following domains: virtual reality (“Virtual Reality*”/”Exergaming*”), mild cognitive impairment (“Cognitive Dysfunction”/”Cognitive Disorder”/”Mild Neurocognitive Disorder*”/”Cognitive Decline*”/”Mild Cognitive Impairment*”/”MCI”), and dementia (“Dementia*”/”Alzheimer Disease*”). The search strategies for all the databases are presented in Supplementary Appendix (Supplementary Table 1).

### 2.2 Eligibility criteria

The following inclusion criteria were utilized in this study. (1) Population: (i) aged ≥ 60 years; (ii) clinically diagnosed with MCI or dementia by a neurologist, psychiatrist, or geriatrician. Diagnostic criteria for MCI included the Petersen criteria, MoCA < 26 (with + 1 adjustment for education ≤ 12 years), or CDR = 0.5. Diagnostic criteria for dementia included DSM-5 Major Neurocognitive Disorder or ICD-10 dementia criteria, education-stratified MMSE cutoffs, or CDR ≥ 1; (2) intervention: any VR-based non-pharmacological intervention. Using computer technology, a three-dimensional environment (which can be immersive, semi-immersive or non-immersive) is generated, aiming to give users a sense of “being there” and enabling them to interact with the environment and the virtual objects within it in real time; (3) comparator: control groups receiving either alternative VR-based interventions, conventional non-pharmacological interventions (such as standard exercise training and reminiscence therapy), or routine clinical care; (4) outcomes: studies reporting ≥ 1 cognitive domain outcome (including global cognition, attention, and executive function) or motor function; and (5) study design: randomized controlled trials.

The exclusion criteria were as follows: (1) studies with inaccessible full-text or unavailable raw data; (2) duplicate publications; (3) case reports; (4) studies that did not evaluate the outcomes of cognitive function or motor function; and (5) research focused mainly on augmented reality or mixed reality technologies.

### 2.3 Study selection and data extraction

The retrieved records were imported into EndNote 21 for duplicate removal. Two investigators independently screened the titles and abstracts against the eligibility criteria. Articles meeting the inclusion criteria underwent full-text review and targeted evaluation. All of the processes were independently conducted; additionally, any discrepancies were resolved via consensus discussion with a third investigator when necessary. Data extraction was independently performed by two researchers via a standardized form adapted from the Cochrane Handbook. The form included study characteristics (such as first author’s name, publication year, title, design, and setting), participant characteristics (such as types of cognitive impairment, living environment, sample size, mean age, and sex ratio), interventions (including types, content, classification of VR immersion levels, interface device, session duration, frequency, and duration), comparators (including category and specific interventions), and outcomes (measurement instruments). The rating scales used for different outcome indicators are listed in sequence in Supplementary Appendix 2 (Supplementary Table 2).

The four VR intervention categories were classified according to the operational definitions detailed in Supplementary Appendix 3. Two investigators independently assigned each included intervention to one of the four predefined categories based on these criteria. Disagreements were resolved through consensus discussion; if no agreement could be reached, a third investigator was consulted as necessary to facilitate consensus discussion.

### 2.4 Assessment of risk of bias

The two authors independently evaluated the included RCTs. The Cochrane risk of bias tool for randomized trials (ROB 2) (Sterne et al., 2019) was used to evaluate the RCTs. The following five domains were assessed: (1) bias arising from the randomization process, (2) bias due to deviations from intended interventions, (3) bias due to missing outcome data, (4) bias in the measurement of the outcome, and (5) bias in the selection of the reported result. The overall risk of bias was classified as low risk of bias, some concern, or high risk of bias. Another author resolved any differences between the two authors who evaluated the risk of bias in the RCTs. The overall certainty of evidence was determined using CINeMA (Confidence in Network Meta-Analysis), a web-based application grounded in the GRADE framework (Nikolakopoulou et al., 2020). The RoB-2 assessments were incorporated into the interpretation of the NMA results and the CINeMA evaluation of certainty, but were not used to weight studies in the statistical synthesis.

### 2.5 Statistical analysis

Prior to the analysis, the outcome measures of this study were classified into distinct categories. The primary outcomes encompassed two dimensions: (1) global cognitive function, assessed using the total scores of the Mini-Mental State Examination (MMSE) or the Montreal Cognitive Assessment (MoCA), reflecting patients’ overall cognitive status; and (2) executive function, analyzed as a representative specific cognitive domain, with assessment tools including the MoCA executive subscores and the Verbal Fluency Test (VFT), among others. The secondary outcome comprised motor function, evaluated using instruments such as the Timed Up and Go Test (TUG) and Gait Speed (GS), which were operationally defined in this study as indicators of lower-extremity mobility and balance control. The detailed correspondence between all outcome indicators and their respective assessment instruments is presented in Supplementary Appendix 2 (Supplementary Table 10).

Initially, we conducted a standard pairwise meta-analysis using the meta package in R to compare the effects of virtual reality-based interventions versus control conditions. A random-effects model was employed to account for potential between-study heterogeneity in intervention effects (Borenstein et al., 2010). All models were fitted using restricted maximum likelihood (REML) (Langan et al., 2019). This method estimates τ^2^ based on the residual likelihood, which automatically accounts for the loss of degrees of freedom from estimating fixed effects, resulting in less biased and more stable estimates, particularly in small samples. In addition, to assess the impact of between-study heterogeneity and to predict the plausible range of intervention effects in future similar studies, we calculated and reported 95% prediction intervals for each pooled estimate (Noma et al., 2023). Post-intervention scores were presented in forest plots, displaying the estimated standardized mean differences (SMDs) with corresponding 95% confidence intervals (CIs) and 95% prediction intervals (PIs). Effect sizes were interpreted using Cochrane guidelines as small (SMD < 0.40), moderate (SMD = 0.40–0.70), or large (SMD > 0.70). Heterogeneity was quantified using the I^2^ statistic, with values of 0%, 25%, 50%, and 75% interpreted as indicating no, low, moderate, and high heterogeneity, respectively. A frequentist framework was adopted for the network meta-analysis. Data analysis was performed using Stata 18.0, as well as the netmeta and metafor packages in R. A multivariate random-effects network meta-analysis framework was implemented using the metafor package in R with restricted maximum likelihood (REML) estimation. Direct evidence from head-to-head randomized controlled trials was combined with indirect evidence via common control arms to generate pooled effect sizes for all pairwise comparisons. We handled the single three-arm trial (Yang et al., 2022) by splitting the shared control group, as recommended by the Cochrane Handbook. This approach preserves all data while avoiding the bias and increased heterogeneity associated with combining intervention arms. These pooled estimates were presented as standardized mean differences (SMDs) with corresponding 95% confidence intervals (CIs) and 95% prediction intervals (PIs) (Shim et al., 2017; Borenstein, 2023). In all analyses, a positive standardized mean difference (SMD) indicated that the intervention group demonstrated greater improvement than the control group did. Owing to the opposing directions of certain assessment scales—where lower scores represent better outcomes for motor function measures—we inverted the effect sizes for these outcomes (multiplied by -1) following calculation. This adjustment ensures a consistent clinical interpretation across all the results, whereby an SMD > 0 uniformly signifies a beneficial effect of the intervention. The specific directionality of all outcome measures, the data harmonization procedures applied, and the assessment tools utilized are detailed in Supplementary Appendix 2.

In the network graph, each node represents an intervention, whereas connecting edges between nodes denote one or more RCTs directly comparing paired interventions (Bhatnagar et al., 2014). Participant weights indicate the node size count receiving each intervention, whereas edge thickness is proportional to the number of studies directly comparing the linked interventions (Puerto Nino and Brignardello-Petersen, 2023). All the VR-based interventions were connected via common comparator interventions within the evidence network, allowing direct and indirect comparisons. In cases where closed loops emerged among treatments, we performed an inconsistency analysis using loop-specific and side-splitting methods to assess the agreement between direct and indirect comparisons.

To evaluate the transitivity assumption (i.e., whether the distribution of effect modifiers is consistent across treatment comparisons), we extracted key covariates, including sample size, mean age, baseline cognitive impairment severity, VR session duration, weekly frequency, total intervention duration, and level of immersion. We then assessed whether the observed differences in the distribution of these covariates across the direct comparison networks were sufficient to violate the transitivity assumption. The structure of the intervention network and the number of available studies for each comparison were deemed suitable for conducting a methodologically sound network meta-analysis. Intervention rankings were evaluated via the surface under the cumulative ranking curve (SUCRA) method (Tan et al., 2023; Lu et al., 2025). SUCRA represents the precise cumulative probability of an intervention being ranked first. Higher SUCRA values indicate superior network rankings, with values ranging from 0% to 100%. A value of 0% denotes the minimal probability of an intervention being optimal, whereas 100% indicates the maximal likelihood. Publication bias was assessed via a comparison-adjusted funnel plot.

To assess intervention effects in specific populations, we conducted separate primary analyses for patients with mild cognitive impairment (MCI) and those with dementia (Borenstein and Higgins, 2013). To explore the causes of heterogeneity further, we also conducted a meta-regression analysis (with the frequency, duration, and duration of VR interventions as covariates) for the primary outcomes. The design-by-treatment interaction model was based on regression analysis, which could explain both the heterogeneity and inconsistency among the studies.

## 3 Results

### 3.1 Identification of relevant studies

**FIGURE 1:**
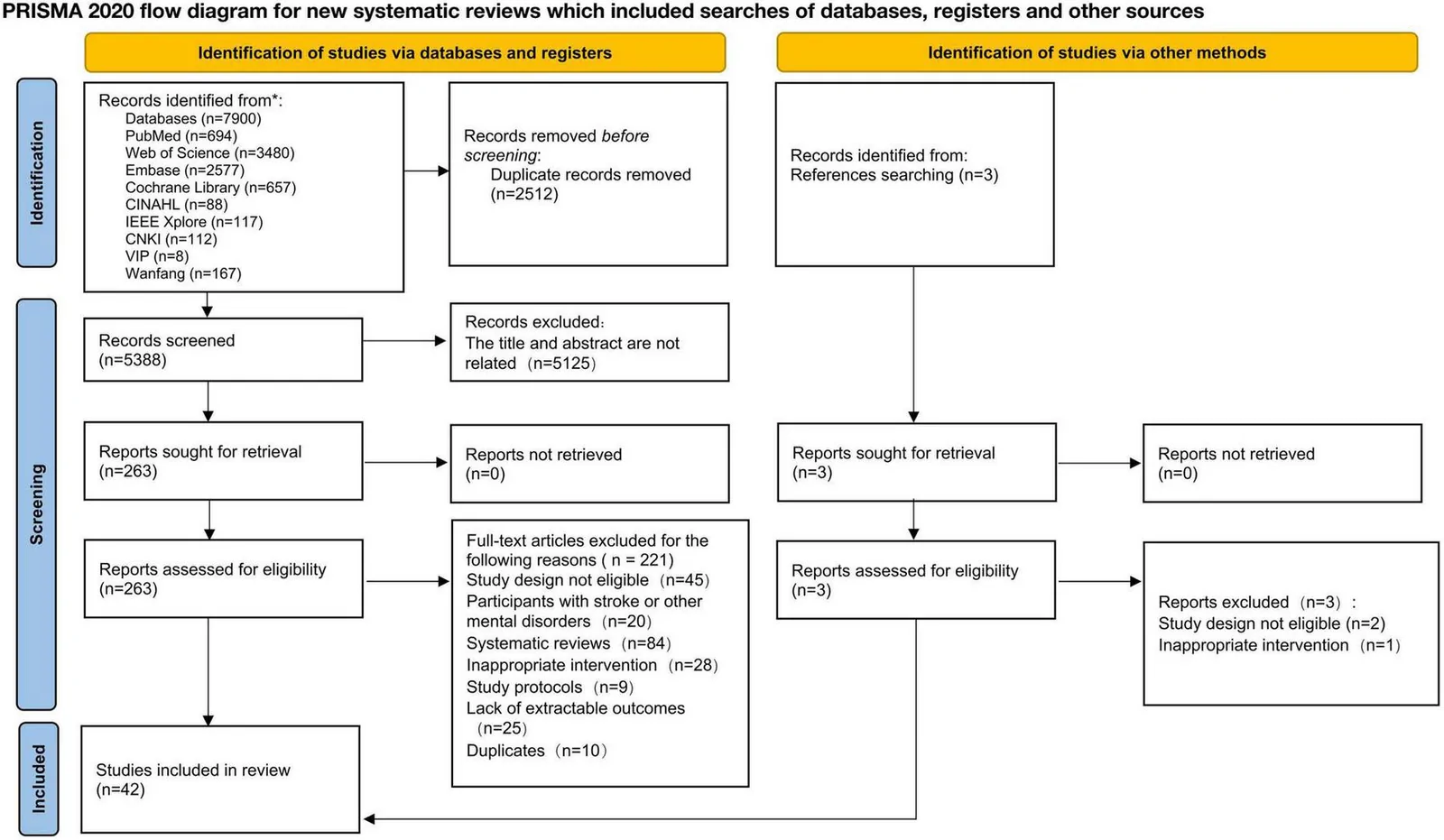
PRISMA flow diagram of the study search and selection process.

An initial literature search yielded 7,900 relevant articles, including 7,613 articles retrieved from English-language databases and 287 articles retrieved from Chinese-language databases. Following the removal of duplicate and clearly irrelevant records, the full texts of 263 articles were assessed for eligibility. A total of 221 articles were excluded because they did not meet the predefined inclusion criteria. Three additional publications were identified via manual screening of reference lists. After a full text evaluation, all of them were excluded as they did not meet the inclusion criteria. Therefore, no additional literature was included. Finally, 42 qualifying studies were selected for inclusion in this NMA, including 31 articles from English-language databases and 11 articles from Chinese-language databases. The detailed selection process is presented in Figure 1. The list of included studies is reported in Supplementary Appendix 5.

### 3.2 Descriptions of the included studies

The different types of VR interventions are classified and listed in Supplementary Appendix 3. These 42 studies involved a total of 1,954 participants in 13 countries across Asia, Europe, South America, North America and Oceania, including 1,612 patients with MCI and 342 patients with dementia. The majority of the participants were aged between 65 and 85 years. The sample sizes ranged from 17 to 80 participants per study. Additionally, the intervention durations spanned 15 to 100 min per session, with frequencies of 1 to 5 weekly sessions reported over periods of 4 to 24 weeks. Among the 42 included studies, 41 were two-arm trials, and one (Yang et al., 2022) was a three-arm trial.

The 42 included studies involved four different non-pharmacological interventions based on VR. These interventions included comprehensive VR cognitive gaming (VR-CCG; 20 studies; *n* = 916 participants), comprehensive VR cognitive gaming and sport training (VR-CCG+SPT; 13 studies; *n* = 549), VR sport training (VR-SPT; 5 studies; *n* = 204), and VR reminiscence therapy (VR-MT; 1 study; *n* = 52). The control conditions were categorized into the following two types: (1) the active control group (ACG), which received traditional interventions analogous to (but differing from) VR-based experimental interventions (including conventional cognitive training versus VR cognitive interventions and traditional exercise versus VR exercise), and (2) the control group (CG), which received standard care, health education, or no intervention. The detailed study characteristics are presented in Supplementary Appendix 3, 4 (Supplementary Table 3).

### 3.3 Risk of bias assessment

Based on the Cochrane Risk of Bias tool (RoB-2), 10 studies were rated as low risk of bias, 30 studies were rated as some concerns, and 2 studies were rated as high risk of bias. The included RCTs demonstrated an acceptable and relatively low risk of bias. Specifically: (1) regarding the randomization process, 10 studies were rated as low risk, 25 studies were categorized as having some concerns due to a lack of reported allocation concealment, and 7 studies were rated as high risk because they failed to report information on both random sequence generation and allocation concealment; (2) regarding deviations from intended interventions, 20 studies that conducted intention-to-treat (ITT) analyses were rated as low risk, 20 studies lacking details on blinding were categorized as having some concerns, and 2 studies that did not blind participants or personnel were rated as high risk; (3) regarding missing outcome data, all 42 studies were rated as low risk, as they either utilized ITT analysis or maintained an attrition rate below 20%; (4) regarding the measurement of the outcome, 40 studies were rated as low risk because they explicitly reported blinding of outcome assessors, whereas 2 studies were categorized as having some concerns due to insufficient details on blinding; (5) regarding the selection of the reported result, all 42 studies were rated as low risk, as they reported outcomes in accordance with the study protocol. Almost all of the trials reported random sequence generation methods, although allocation concealment procedures remained undescribed in most of the studies, which may introduce potential selection bias. The detailed risk of bias assessments for the included studies are shown in Supplementary Figures 1, 2.

### 3.4 Traditional pairwise meta-analysis

Compared with the CG, VR-CCG (*n* = 11; SMD = 0.52; 95% CI [0.19 to 0.85]; 95% PI [−0.53 to 1.58]; I^2^ = 64.6%) and VR-CCG+SPT (*n* = 6; SMD = 0.66; 95% CI [0.08 to 1.24]; 95% PI [−1.14 to 2.46]; I^2^ = 76.6%) had positive effects on cognitive function in older adults with cognitive impairments. The direct comparison results are presented in Supplementary Figures 3–5. Compared with ACG, VR-CCG+SPT (*n* = 5; SMD = 0.56; 95% CI [0.28 to 0.83]; 95% PI [0.17 to 0.94]; I^2^ = 1.9%) had positive effects on cognitive function. Other interventions demonstrated no significant effects on cognitive function in older adults with cognitive impairments. The direct comparison results are presented in Supplementary Figures 6–8. Moreover, our stratified analysis by diagnostic category (MCI and dementia) revealed some inconsistent results. Compared with the CG, VR-CCG (*n* = 8; SMD = 0.48; 95% CI [0.03 to 0.93]; 95% PI [−0.93 to 1.90]; I^2^ = 74.3%) and VR-CCG+SPT (*n* = 6; SMD = 0.66; 95% CI [0.08 to 1.24]; 95% PI [−1.14 to 2.46]; I^2^ = 76.6%) had positive effects on cognitive function in older adults with MCI. Compared with the ACG, VR-CCG+SPT (*n* = 5; SMD = 0.56; 95% CI [0.28 to 0.83]; 95% PI [0.17 to 0.94]; I^2^ = 1.9%) had positive effects on cognitive function in older adults with MCI. The other interventions did not significantly affect cognitive function in older adults with MCI. The direct comparison results are presented in Supplementary Figures 9–12. Compared with the CG, the VR-CCG (*n* = 3; SMD = 0.57; 95% CI [0.19 to 0.94]; 95% PI [−0.26 to 1.39]; I^2^ = 0.0%) had positive effects on cognitive function in older adults with dementia. The direct comparison results are presented in Supplementary Figure 13.

Compared with the CG, all the interventions demonstrated no significant effects on executive function in older adults with cognitive impairments. The direct comparison results are presented in Supplementary Figures 14, 15. Compared with ACG, the VR-CCG+SPT (*n* = 4; SMD = 0.62; 95% CI [0.23 to 1.00]; 95% PI [−0.19 to 1.42]; I^2^ = 20.8%) had positive effects on executive function. The direct comparison results are presented in Supplementary Figures 16, 17. Owing to the limited number of studies, we only conducted a main meta-analysis of patients with MCI. Compared with the CG, none of the interventions significantly affected executive function in older adults with MCI. The direct comparison results are presented in Supplementary Figures 18, 19. Compared with ACG, the VR-CCG+SPT (*n* = 4; SMD = 0.62; 95% CI [0.23 to 1.00]; 95% PI [−0.19 to 1.42]; I^2^ = 20.8%) had positive effects on executive function in older adults with MCI. The direct comparison results are presented in Supplementary Figures 20, 21.

Compared with the CG, VR-CCG+SPT (*n* = 4; SMD = 0.54; 95% CI [0.09 to 0.99]; 95% PI [−0.49 to 1.57]; I^2^ = 8.9%) and VR-SPT (*n* = 3; SMD = 0.54; 95% CI [0.21 to 0.87]; 95% PI [−0.18 to 1.26]; I^2^ = 0.0%) had positive effects on motor function in older adults with cognitive impairments. The direct comparison results are presented in Supplementary Figures 22, 23. Compared with the ACG, none of the interventions significantly affected motor function in older adults with cognitive impairments. The direct comparison results are presented in Supplementary Figure 24. Owing to the limited number of studies, we only conducted a main meta-analysis on patients with MCI. Compared with the CG, the VR-SPT (*n* = 2; SMD = 0.47; 95% CI [0.10 to 0.84]; 95% PI [−1.92 to 2.85]; I^2^ = 0.0%) had positive effects on motor function in older adults with MCI. The other interventions did not significantly affect motor function in older adults with MCI. The direct comparison results are presented in Supplementary Figures 25, 26. Compared with the ACG, none of the interventions demonstrated significant effects on motor function in older adults with MCI. The direct comparison results are presented in Supplementary Figure 27.

Meta-regression analyses were performed separately for cognitive, executive, and motor functions to examine the potential moderating effects of intervention frequency, session duration, total intervention duration, and immersion level. The results indicated that none of these covariates significantly moderated the effects of VR interventions on cognitive or executive functions. In contrast, the level of immersion was identified as a significant effect modifier for motor function outcomes. The complete results are provided in Supplementary Appendix 8.

### 3.5 Network meta-analysis

#### 3.5.1 Primary outcomes

We evaluated the transitivity assumption by comparing the distribution of key covariates across different VR intervention groups (Supplementary Appendix 9). Baseline characteristics, including age and sex distribution, were generally comparable across study groups. Meta-regression revealed that none of the examined covariates significantly moderated the effects on global cognition or executive function (*p* > 0.05), whereas immersion level significantly moderated motor function outcomes. Additionally, no statistically significant inconsistency was detected across any outcome network (*p* > 0.05). Taken together, these findings suggest that the indirect comparisons in all three outcome networks have an acceptable basis for transitivity. However, given the significant moderating effect of immersion on motor outcomes and the limited number of studies for certain interventions (e.g., VR-SPT), the ranking results should be interpreted as exploratory and applied cautiously in clinical practice.

**FIGURE 2:**
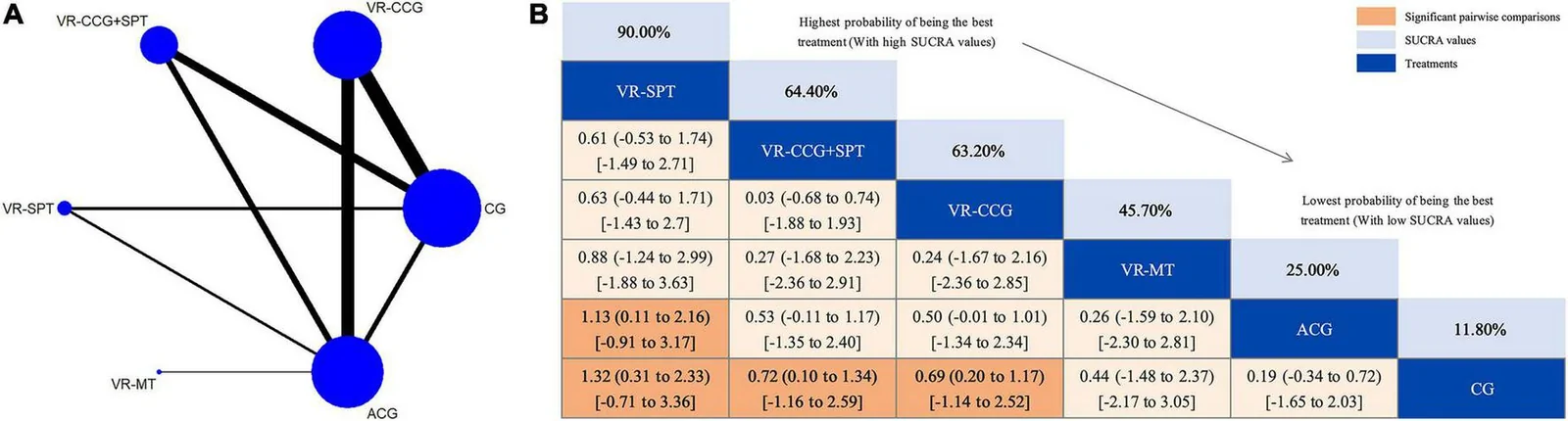
Network graph **(A)** and relative effect sizes combined with SUCRA values of different interventions according to network meta-analysis **(B)** for global cognitive function. In panel **(A)**, node size reflects the number of participants, and edge thickness reflects the number of direct comparisons. In panel **(B)**, the 95% confidence interval (indicated by parentheses) and the 95% prediction interval [indicated by square brackets] are presented. A relative effect value higher than 0 favors the column-defining intervention, whereas a value lower than 0 favors the row-defining intervention. SUCRA values indicate the probability of each intervention being ranked as the best. VR-CCG, VR Comprehensive Cognitive Game; VR-CCG+SPT, VR Comprehensive Cognitive Game+Sport Training; VR-SPT, VR Sport Training; VR-MT, VR Reminiscence Therapy; CG, control group; ACG, active control group; SUCRA, surface under the cumulative ranking curve; PI, prediction interval.

For global cognitive function, 39 studies involving a total of 1,754 participants were included. Among these studies, 20 (Man et al., 2012; Song, 2018; Amjad et al., 2019; Yang et al., 2019; Manenti et al., 2020; Park J. H. et al., 2020; Park J. S. et al., 2020; Thapa et al., 2020; Xie et al., 2020; Xu et al., 2020; Kang et al., 2021; Oliveira et al., 2021; Xue and Qin, 2021; Arshad et al., 2023; Ding, 2023; Goumopoulos et al., 2023; Lim et al., 2023) assessed VR comprehensive cognitive gaming (VR-CCG), 11 (Hagovská and Olekszyová, 2016; Schwenk et al., 2016; Delbroek et al., 2017; Hu et al., 2018; Choi and Lee, 2019; Liao et al., 2019; Li et al., 2021; Sun et al., 2021b; Buele et al., 2024; Li et al., 2024; Ip et al., 2025) assessed VR comprehensive cognitive gaming combined with exercise training (VR-CCG+SPT), four (Padala et al., 2012; Sertel and Uğur, 2020; Sun et al., 2021a; Liu et al., 2022) assessed VR exercise training (VR-SPT) and one (Tominari et al., 2021) assessed VR reminiscence therapy (VR-MT). The network graph (Figure 2A) illustrates all of the available comparisons among the included studies. The global inconsistency test and local inconsistency test revealed no significant inconsistency, with detailed results presented in Supplementary Figure 28 in Supplementary Appendix 7.2.1. The relative effects of the interventions are presented in Figure 2B. Specifically, the comparisons of VR-SPT versus CG (SMD = 1.32; 95% CI [0.31 to 2.33]; 95% PI [−0.71 to 3.36]), VR-CCG+SPT versus CG (SMD = 0.72; 95% CI [0.10 to 1.34]; 95% PI [−1.16 to 2.59]) and VR-CCG versus CG (SMD = 0.69; 95% CI [0.20 to 1.17]; 95% PI [−1.14 to 2.52]) were statistically significant; moreover, the comparisons of VR-SPT versus ACG (SMD = 1.13; 95% CI [0.11 to 2.16]; 95% PI [−0.91 to 3.17]) were statistically significant. VR intervention rankings based on SUCRA values and cumulative probability plots are shown in Figure 2B and Supplementary Table 13 in Supplementary Appendix 7.2.1 and Supplementary Figure 29. In terms of the SUCRA values, VR-SPT was ranked as the most effective intervention, followed by VR-CCG+SPT, VR-CCG, VR-MT, ACG and CG. Preliminary stratified analyses by patient group (i.e., patients with mild cognitive impairment vs. those with dementia) revealed discrepancies in the findings. For patients with MCI, VR-SPT was still the most effective intervention, with a SUCRA value of 99.6% (Supplementary Appendix 7.2.2). Specifically, the comparisons of VR-SPT versus CG (SMD = 2.90; 95% CI (1.50 to 4.31); 95% PI [0.68 to 5.12]), VR-SPT versus ACG (SMD = 2.76; 95% CI (1.34 to 4.18); 95% PI [0.53 to 4.99]), VR-CCG versus CG (SMD = 0.67; 95% CI (0.13 to 1.21); 95% PI [−1.13 to 2.47]), and VR-CCG+SPT versus CG (SMD = 0.69; 95% CI (0.04 to 1.34); 95% PI [−1.15 to 2.52]) were statistically significant. However, for patients with dementia, VR-CCG+SPT was deemed the most effective intervention measure with the highest probability (SUCRA = 84.3%), far exceeding that of VR-SPT, with a SUCRA value of 16.6% (Supplementary Appendix 7.2.3). Specifically, the comparisons of VR-CCG+SPT versus CG (SMD = 0.77; 95% CI (0.14 to 1.41); 95% PI [0.06 to 1.49]) and VR-CCG versus CG (SMD = 0.67; 95% CI (0.26 to 1.08); 95% PI [0.15 to 1.20]) were statistically significant. It should be noted that, given the limited evidence base (e.g., only one study for VR-MT and four for VR-SPT), the SUCRA ranking results are subject to substantial uncertainty and should therefore be interpreted with caution.

**FIGURE 3:**
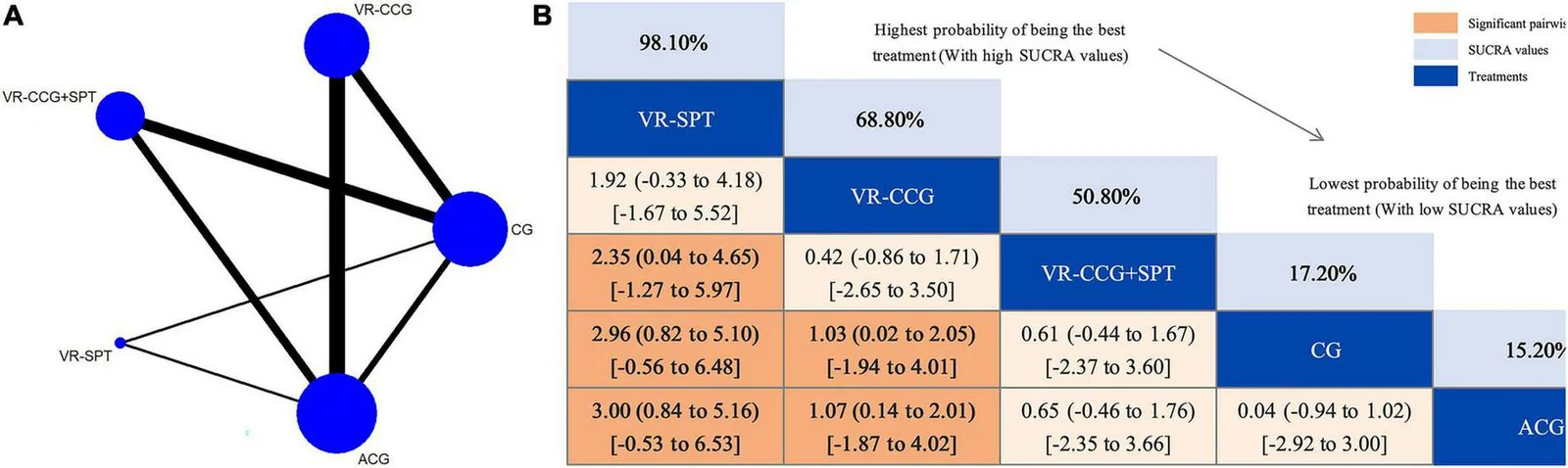
Network graph **(A)** and relative effect sizes combined with SUCRA values of different interventions according to network meta-analysis **(B)** for executive function. In panel **(A),** node size reflects the number of participants, and edge thickness reflects the number of direct comparisons. In panel **(B),** the 95% confidence interval (indicated by parentheses) and the 95% prediction interval [indicated by square brackets] are presented. A relative effect value higher than 0 favors the column-defining intervention, whereas a value lower than 0 favors the row-defining intervention. SUCRA values indicate the probability of each intervention being ranked as the best. VR-CCG, VR Comprehensive Cognitive Game; VR-CCG+SPT, VR Comprehensive Cognitive Game+Sport Training; VR-SPT, VR Sport Training; CG, control group; ACG, active control group; SUCRA, surface under the cumulative ranking curve; PI, prediction interval.

With respect to executive function, 26 studies involving 1,158 participants were included. Among these studies, 12 (Song, 2018; Amjad et al., 2019; Yang et al., 2019; Park J. H. et al., 2020; Park J. S. et al., 2020; Thapa et al., 2020; Oliveira et al., 2021; Xue and Qin, 2021; Yang et al., 2022; Arshad et al., 2023; Goumopoulos et al., 2023; Lim et al., 2023) assessed VR comprehensive cognitive gaming (VR-CCG), nine (Schwenk et al., 2016; Delbroek et al., 2017; Hu et al., 2018; Choi and Lee, 2019; Liao et al., 2020; Li et al., 2023; Buele et al., 2024; Li et al., 2024; Ip et al., 2025) assessed VR comprehensive cognitive gaming combined with exercise training (VR-CCG+SPT), three studies assessed active control group (ACG), and two (Sun et al., 2021a; Liu et al., 2022) assessed VR exercise training (VR-SPT). The global executive function network graph (Figure 3A) illustrates all of the available comparisons among the included studies. The global inconsistency test and local inconsistency test revealed no significant inconsistency, with detailed results presented in Supplementary Figure 41 in Supplementary Appendix 7.2.4. The relative effects of the interventions are presented in Figure 3B. Specifically, the comparisons of VR-SPT versus CG (SMD = 2.96; 95% CI (0.82 to 5.10); 95% PI [−0.56 to 6.48]) and VR-CCG versus CG (SMD = 1.03; 95% CI (0.02 to 2.05); 95% PI [−1.94 to 4.01]) were statistically significant; moreover, the comparisons of VR-SPT versus ACG (SMD = 3.00; 95% CI (0.84 to 5.16); 95% PI [−0.53 to 6.53]), VR-CCG versus ACG (SMD = 1.07; 95% CI (0.14 to 2.01); 95% PI [−1.87 to 4.02]), and VR-SPT versus VR-CCG+SPT (SMD = 2.35; 95% CI (0.04 to 4.65); 95% PI [−1.27 to 5.97]) were statistically significant. VR intervention rankings based on SUCRA values and cumulative probability plots are shown in Figure 3B and Supplementary Table 16 in Supplementary Appendix 7.2.4 and Supplementary Figure 42. In terms of the SUCRA values, VR-SPT was ranked as the most effective intervention, followed by VR-CCG, VR-CCG+SPT, CG and ACG. Among all the studies investigating executive functions, only one was conducted specifically in patients with dementia. Consequently, we only conducted the analysis on patients with MCI. For patients with MCI, VR-SPT was still the most effective intervention (SMD = 2.98; 95% CI (0.78 to 5.19); 95% PI [−0.65 to 6.61]), with a SUCRA value of 97.7 (Supplementary Appendix 7.2.5). Specifically, the comparisons of VR-SPT versus CG (SMD = 2.98; 95% CI (0.78 to 5.19); 95% PI [−0.65 to 6.61]), VR-CCG versus CG (SMD = 1.12; 95% CI (0.00 to 2.24); 95% PI [−1.97 to 4.21]), VR-SPT versus ACG (SMD = 2.98; 95% CI (0.76 to 5.21); 95% PI [−0.66 to 6.63]) and VR-CCG versus ACG (SMD = 1.12; 95% CI (0.14 to 2.10); 95% PI [−1.92 to 4.16]) were statistically significant. However, the two aforementioned conclusions are drawn from only two studies (Sun et al., 2021a; Liu et al., 2022), and the evidence is of lower certainty due to the very wide confidence intervals, which may render the results unstable. Thus, the pronounced therapeutic benefits of VR-SPT for improving executive function in the studied population, particularly in patients with both MCI and AD (SMD = 2.96; 95% CI (0.82 to 5.10); 95% PI [−0.56 to 6.48]) and in those with MCI alone (SMD = 2.98; 95% CI (0.78 to 5.19); 95% PI [−0.65 to 6.61]), must be interpreted with great caution.

#### 3.5.2 Secondary outcomes

**FIGURE 4:**
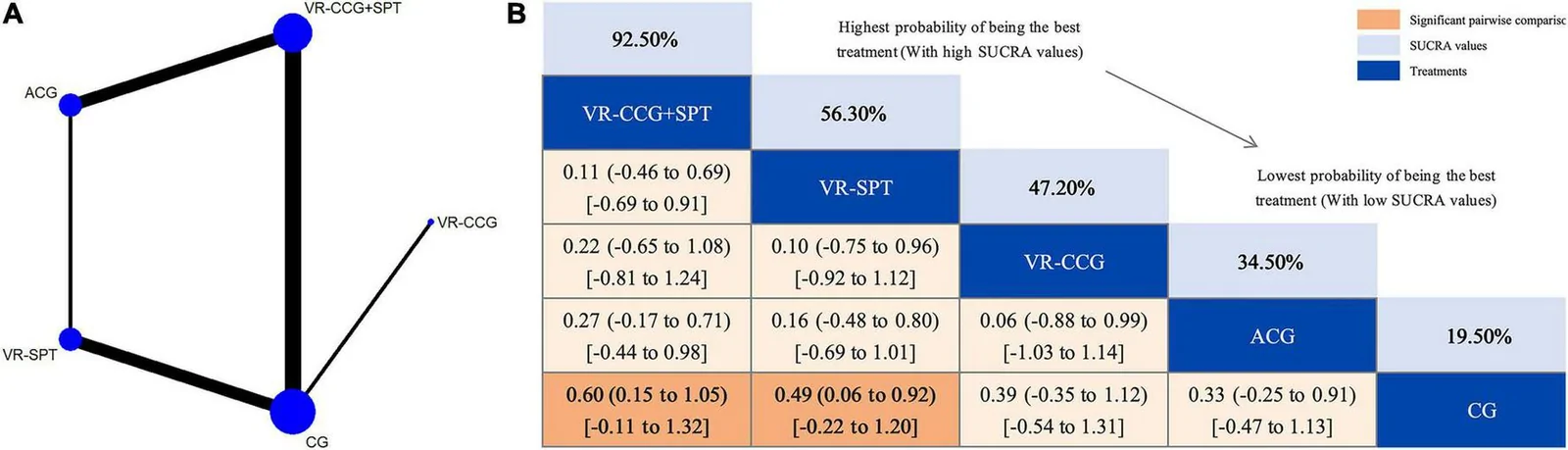
Network graph **(A)** and relative effect sizes combined with SUCRA values of different interventions according to network meta-analysis **(B)** for motor function. In panel **(A),** node size reflects the number of participants, and edge thickness reflects the number of direct comparisons. In panel **(B)**, the 95% confidence interval (indicated by parentheses) and the 95% prediction interval [indicated by square brackets] are presented. A relative effect value higher than 0 favors the column-defining intervention, whereas a value lower than 0 favors the row-defining intervention. SUCRA values indicate the probability of each intervention being ranked as the best. VR-CCG, VR Comprehensive Cognitive Game; VR-CCG+SPT, VR Comprehensive Cognitive Game+Sport Training; VR-SPT, VR Sport Training; CG, control group; ACG, active control group; SUCRA, surface under the cumulative ranking curve; PI, prediction interval.

For motor function, 12 studies involving 515 participants were selected. Among these studies, seven (Hughes et al., 2014; Hagovská and Olekszyová, 2016; Schwenk et al., 2016; Delbroek et al., 2017; Choi and Lee, 2019; Sun et al., 2021b; Ip et al., 2025) assessed comprehensive VR cognitive gaming combined with exercise training (VR-CCG+SPT), four (Padala et al., 2012; Hsieh et al., 2018; Sertel and Uğur, 2020; Sun et al., 2021a) assessed VR exercise training (VR-SPT), and one (Thapa et al., 2020) assessed comprehensive VR cognitive gaming (VR-CCG). The global motor function network graph (Figure 4A) illustrates all of the available comparisons among the included studies. The global inconsistency test and local inconsistency test revealed no significant inconsistency, with detailed results presented in Supplementary Figure 49 in Supplementary Appendix 7.2.6. The relative effects of the interventions are presented in Figure 4B. Specifically, the comparisons of VR-CCG+SPT versus CG (SMD = 0.60; 95% CI (0.15 to 1.05); 95% PI [−0.11 to 1.32]) and VR-SPT versus CG (SMD = 0.49; 95% CI (0.06 to 0.92); 95% PI [−0.22 to 1.20]) demonstrated statistical significance. VR intervention rankings based on SUCRA values and cumulative probability plots are shown in Figure 4B and Supplementary Table 18 in Supplementary Appendix 7.2.6 and Supplementary Figure 50. In terms of the SUCRA values, VR-CCG+SPT was the most effective intervention, followed by VR-SPT, VR-CCG, ACG and CG. However, owing to the limited evidence and the small number of studies for each intervention node, the precision of the rankings is highly uncertain, and caution is warranted in their interpretation. Owing to the limited number of studies (*n* = 3) involving patients with dementia, only those with MCI were eligible for inclusion in the primary analysis. In the analysis of patients with MCI, none of the interventions significantly affected motor function (Supplementary Appendix 7.2.7).

Across the three outcome measures, the 95% prediction intervals (PIs) for most comparisons were consistently wider than their corresponding 95% confidence intervals (CIs) and often encompassed the null value (SMD = 0), despite point estimates suggesting potential benefits. Taking global cognitive function as an example, although VR-SPT, VR-CCG+SPT, and VR-CCG appeared to confer advantages over CG on average, the wide PIs indicated that future trials conducted in different populations or contexts could yield only trivial effects, no clear difference, or even unfavorable outcomes. This pattern of wide PIs crossing the null was also evident for executive function and motor function. Collectively, these findings highlight that between-study heterogeneity and variation in implementation conditions may result in substantially divergent effects when VR interventions are applied in real-world practice.

#### 3.5.3 Strength of evidence

The certainty of evidence for the network meta-analysis results was assessed using the CINeMA framework. All included studies were randomized controlled trials (RCTs), and the quality of evidence was evaluated according to the Cochrane Handbook, with each potential source of bias rated as low risk, high risk, or some concerns. Across all outcomes, the certainty of evidence for all comparisons involving direct evidence was rated as very low, primarily due to within-study bias, imprecision, and heterogeneity. All evidence derived solely from indirect comparisons was also rated as very low. The detailed certainty ratings for all comparisons are presented in Supplementary Appendix 10.

### 3.6 Publication bias

Visual inspection of the comparison-adjusted funnel plots revealed no discernible asymmetry, thus indicating that there was no evidence of publication bias. Comparison-adjusted funnel plots are presented in Supplementary Figures 30, 35, 40, 43, 48, 51 and 56.

## 4 Discussion

### 4.1 Summary and interpretation of findings

This study compared the relative effects of various VR-based interventions on global cognition, executive function, and motor function in individuals with MCI or dementia. To our knowledge, no NMA has compared various VR-based interventions for assessing cognitive and motor function in older adults with MCI or dementia. Therefore, this NMA represents the first effort to examine the relative effects of VR-based interventions on both global and specific cognitive domains, as well as motor function. VR-based interventions have been confirmed to have beneficial effects on global cognition, executive function, and motor function in individuals with MCI or dementia. Our findings also confirm the beneficial effects of various VR interventions on cognitive and motor functions. The results of this analysis suggest that the VR-SPT may be one of the most promising interventions for mitigating global cognitive and executive function decline in patients with mild cognitive impairment or dementia. In contrast, VR-CCG+SPT appears most effective for preserving motor function in this population. Analyses confined to specific patient populations further indicated that VR-SPT was the most effective intervention for improving global cognition and executive function in patients with MCI, whereas VR-CCG+SPT demonstrated superior efficacy for global cognitive function in patients with dementia.

Our results revealed that the majority of comparisons across all outcome networks were informed by only a small number of studies, a substantial proportion of which were rated as having some concerns or a high risk of bias in at least one domain of the RoB-2 tool. In the complementary frequentist NMAs, 95% prediction intervals (PIs) were consistently wide and frequently encompassed the null value, despite point estimates suggesting potential benefits. Furthermore, all network comparisons were graded as very low certainty according to the CINeMA assessment. Taken together, these findings indicate that our estimates should be interpreted as uncertain average effects rather than precise predictions. Of particular concern were biases arising from the within-study bias, imprecision, and heterogeneity, which likely increased the uncertainty in the network estimates and were key factors contributing to the very low certainty ratings in our CINeMA evaluation.

VR-SPT has received considerable attention owing to its beneficial effects on cognitive function (global cognition and executive function) in older adults with cognitive impairments. In particular, it is regarded as the most promising intervention measure among patients with MCI. In recent years, the number of studies on VR-SPT has continued to increase. Our findings align with those of previous systematic reviews (Baldimtsi et al., 2023; Chan et al., 2024), thus confirming that VR-SPT improves cognitive function in this specific patient population. These positive outcomes may be explained by several mechanisms. First, exercise induces beneficial metabolic effects by increasing brain- derived neurotrophic factor (BDNF) levels and enhancing hippocampal plasticity (Tsai et al., 2019; Broadhouse et al., 2020), thereby potentiating the brain’s neuroplastic potential (Barha et al., 2017) and accelerating learning during task execution. Second, exercise stimulates the hypothalamic–pituitary–adrenal (HPA) axis (Duclos and Tabarin, 2016), thus increasing cortisol levels to facilitate memory and learning (Broadhouse et al., 2020). Additionally, an increase in muscle strength or mass due to exercise is associated with better cognitive performance (Feter et al., 2023). Finally, exercise may beneficially modulate proinflammatory cytokines, including interleukin-1β (IL-1β), and interleukin-15 (IL-15), thereby exerting positive effects on the brain and cognition (Kong et al., 2025). The flexibility of VR enables safe training in scenarios that are considered potentially hazardous in real-world settings (Cooper et al., 2005). Furthermore, VR systems can be adapted to participants’ physical capabilities (Gourlay et al., 2000), thereby resulting in VR-SPT being a suitable intervention method for individuals with MCI or dementia. Notably, the current evidence base for VR-SPT regarding executive function is remarkably weak, supported by only two studies. Therefore, the conclusion that VR-SPT improves executive function in patients with cognitive impairment should be regarded as exploratory and awaits verification in future studies with larger sample sizes and more rigorous methodological designs.

With respect to executive function, significant effects were observed for both the VR-SPT and the VR-CCG, with the VR-SPT demonstrating superior efficacy compared with the VR-CCG. Research has indicated that the most significant benefits of exercise training interventions in older adults may manifest in executive function (Colcombe and Kramer, 2003), with positive effects being observed across multiple domains (including memory, shifting, and inhibition), which is consistent with the results of prior systematic reviews (Northey et al., 2018; Wilke et al., 2019). The underlying mechanisms of these effects are similar to those for improving cognitive function; specifically, VR exercise induces BDNF release (Anderson-Hanley et al., 2012) and exercise stimulates dopamine release in the striatal–prefrontal circuitry (Bastioli et al., 2022). These effects may explain the enhanced information processing capacity and specific improvements in the shifting and inhibitory control components of executive function.

However, importantly, while VR-SPT was ranked highest for improving cognitive function and executive function, this conclusion is tempered by the limited evidence base—being derived from only two studies—and the very wide confidence intervals observed. Therefore, these promising findings should be interpreted with caution. At present, few RCTs examining executive function in older adults with cognitive impairment have been identified. Therefore, future multi-center RCTs with larger sample sizes are warranted to address this evidence gap.

For motor function, significant effects were observed for both the VR-CCG+SPT and the VR-SPT. However, VR-CCG+SPT demonstrated more favorable effects on improving motor function in older adults with cognitive impairment. Previous studies have indicated (Li et al., 2024) that VR-CCG+SPT positively influences global cognition. Specifically, the participants exhibited progressively increased accuracy and reduced task completion times during motor tasks as the intervention duration increased, thus confirming enhanced motor function, which was consistent with our findings. The following mechanisms may explain these effects. First, during VR-CCG+SPT, the high spatiotemporal congruence between visual targets and spatialized auditory cues activates multiple sensory brain regions, including the thalamus, cerebellum, and inferior parietal lobe. This activation enhances early sensory integration and processing efficiency, thereby accelerating decision-making and response execution. Auditory cues not only provide spatial orientation but also guide visual attention allocation through cross-modal correspondences, such as pitch–position associations (Alwashmi et al., 2024). As participants internalize these correspondences, they form an automatic “auditory–visual–motor” loop, facilitating the transfer of learning effects to untrained tasks and promoting cognitive improvement (Kim and Lee, 2022); this is also the main reason why VR intervention without movement can still significantly improve patients’ cognitive and executive functions. At the motor level, auditory spatial information contributes directly to movement vector computation. Enhanced functional connectivity between the inferior parietal lobe and pre-motor cortex supports the efficient conversion of spatial perception into motor commands, enabling rapid mapping from “auditory localization” to “motor orientation” (Wu et al., 2024). Through repeated exposure to multisensory VR inputs coupled with varied motor tasks, participants establish an integrated “perception–cognition–action” neural pathway, ultimately optimizing task performance in the virtual environment (Riva et al., 2021). Second, VR-CCG+SPT promotes social interaction (Szczepocka et al., 2024) and enhances active communication abilities (Kalantari et al., 2023) among older adults with cognitive impairments. The gamified design of VR-CCG enhances participant motivation. Its real-time, personalized task adjustment feature enables dynamic difficulty calibration, thereby maintaining an appropriate challenge threshold for individual users. This process establishes a positive feedback loop that can improve treatment adherence, particularly among long-term care residents. This mechanism may explain the superior efficacy of combined VR-CCG+SPT compared with VR-SPT alone.

Meta-regression analysis revealed the level of VR immersion as a significant effect modifier for motor outcomes, with higher immersion levels associated with greater intervention efficacy. These findings align with the work of Ren et al. (2024), who reported that immersive VR-based training has stronger effects on cognitive and motor functions than non-immersive VR training does. Thus, researchers should consider these factors in future studies. Currently, research in this domain is limited. Future large-sample RCTs utilizing standardized protocols are needed to verify the optimal session duration, frequency, treatment period, and efficacy of VR-CCG+SPT.

This study provides initial evidence to inform the selection of personalized intervention strategies in clinical practice. Our network meta-analysis results indicate that VR-SPT demonstrated superior efficacy for improving global cognition and executive function in patients with mild cognitive impairment (MCI), whereas VR-CCG+SPT may be more beneficial for patients with dementia. However, the relationships between key patient baseline characteristics—such as cognitive impairment severity, physical function, and socioeconomic status—and the effectiveness of different VR interventions remain unclear due to a lack of evidence. Future research should prioritize the systematic collection and analysis of these multi-dimensional patient data to establish an empirical basis for developing truly personalized VR rehabilitation protocols. Second, regarding the optimal dosing parameters for VR interventions, our meta-regression analysis revealed that intervention frequency, session duration, and total program length did not significantly impact the improvement in cognitive, executive, or motor functions (*p* > 0.05).

These findings suggest that VR interventions may have a relatively broad effective dosing range for enhancing cognitive and motor functions in older patients with cognitive impairment. Even short-term interventions (e.g., 4–8 weeks) may yield clinically significant effects. These findings align with the work of Ren et al. (2024), who reported that longer intervention cycles do not necessarily yield superior outcomes for this population. A potential neurophysiological mechanism is that VR, through its high-intensity immersion and multi-sensory stimulation, can rapidly engage brain regions critical for learning and functional reorganization, such as the premotor and parietal cortices, thereby inducing substantial short-term neuroplastic changes. Most trials in this analysis reported only immediate post-intervention data, with limited long-term follow-up assessments. Existing evidence indicates that follow-up periods in VR intervention studies are generally short, typically ranging from 1 to 3 months and rarely exceeding 6 months, resulting in uncertain long-term effect sustainability and a potential risk of effect attenuation over time (Wei et al., 2025). Consequently, future research should prioritize the development of time-and cost-efficient short-term intervention protocols, shifting the focus from pursuing the “maximum dose” to identifying strategies such as maintenance training that can consolidate short-term therapeutic gains.

Further elaboration on the interpretation of prediction intervals reveals that the observed benefits of VR interventions may vary considerably across diverse populations. In most comparisons, the 95% prediction intervals were wide and frequently crossed the null value. This pattern suggests that, although certain VR interventions demonstrated average benefits in improving cognitive, executive, and motor functions among patients with cognitive impairment, their implementation in new populations or settings may yield smaller effects or even no apparent benefits. This underscores the necessity for cautious adaptation, rigorous monitoring, and comprehensive evaluation when implementing VR interventions.

This network meta-analysis provides important insights for clinical practice and future research. First, in clinical settings, VR-SPT may positively influence cognitive outcomes in patients with cognitive impairment (particularly those with MCI), whereas VR-CCG+SPT demonstrates superior efficacy in enhancing motor function. For patients with dementia, VR-CCG+SPT also showed a potential benefit for global cognitive function, though this finding is based on a limited number of studies and requires confirmation in future larger-scale trials. Therefore, these interventions can be regarded as adjunctive therapies for patients with cognitive dysfunction. Further analysis of the prediction intervals indicates that the observed benefits of VR interventions may vary substantially across different populations. In most comparisons, the 95% prediction intervals were wide and frequently crossed the null value. This pattern suggests that although certain VR interventions demonstrated average improvements in cognitive and motor functions among patients with cognitive impairment, their effects may be smaller or even lack apparent benefits when implemented in new populations or settings. This underscores the necessity for cautious adaptation, rigorous monitoring, and comprehensive evaluation when implementing VR interventions. This is particularly true for the effect of VR-SPT on executive function, where the limited reliability of the available evidence and substantial heterogeneity largely explain the particularly wide prediction interval, underscoring the need for cautious interpretation. Second, although our findings demonstrate the beneficial effects of VR interventions, the body of evidence for MCI and AD populations remains limited. Therefore, large-scale, well-designed RCTs are needed to strengthen direct evidence regarding the comparative effectiveness of different VR interventions on cognitive and motor outcomes. Third, the optimal dosing parameters—including frequency, session duration, intervention period, and effect sustainability—for both VR-CCG+SPT and VR-SPT require further investigation and should be a focus of future research. Finally, future efforts should prioritize interdisciplinary collaboration to systematically develop novel VR-based interventions beyond the existing four modalities, improve current approaches, and explore the combined effects of different intervention strategies.

### 4.2 Limitations

This network meta-analysis has several limitations: (1) all of the available VR interventions targeting cognitive and motor functions in individuals with MCI or dementia were identified, but some interventions were excluded due to unavailable outcome data, which severely limited the available data; (2) the limited number of included studies inevitably contributed to uncertainty in the results. Specifically, the extremely sparse data for certain VR interventions led to insufficient direct evidence for several comparisons, which may have reduced the precision of the effect estimates. Consequently, these findings should be interpreted with caution; (3) follow-up data were only reported in a few studies, with only postintervention data being extracted, thereby precluding assessments of effect sustainability for cognitive, executive, and motor functions after intervention cessation; (4) the transitivity assumption underlying the network meta-analysis cannot be empirically verified. While we conducted a descriptive assessment of transitivity, residual differences across studies persist, such as the degree of VR immersion. Furthermore, substantial statistical heterogeneity suggests the potential influence of other unmeasured effect modifiers, including variations in randomization procedures, blinding, subtle baseline cognitive differences, and diverse care environments. Although we performed stratified analyses by diagnostic category and meta-regression, the limited number of studies in certain groups constrained their statistical power. Consequently, considerable caution is warranted when interpreting the intervention rankings; (5) most of the included studies did not report the blinding procedures related to randomization, which may introduce a potential risk of bias. Owing to within-study bias and imprecision, the majority of network comparisons were rated as having very low certainty of evidence. Furthermore, the prediction intervals (PIs) for most comparisons were generally wide, suggesting that the true effects may differ substantially from our estimates. Therefore, both the SUCRA rankings and effect estimates derived from this network meta-analysis should be interpreted with considerable caution; (6) a key methodological limitation of this study is that data from trials reporting multiple outcome measures were incorporated into separate, independent networks. This has theoretically affected the assumption of independence and potentially introduced bias into the pooled estimates. Therefore, the results of the network comparisons should be interpreted with caution; (7) although we did not detect significant funnel plot asymmetry, given that our literature search was primarily confined to Chinese and English databases, there remains a potential risk of language bias, as well as publication bias stemming from unpublished negative findings; (8) of note, nearly all included studies failed to systematically assess participants’ baseline familiarity with VR technology, thereby limiting a comprehensive analysis of its clinical feasibility. Future research should adopt standardized instruments to report VR tolerability and user experience, and evaluate patient-level factors—such as prior technological familiarity—on intervention outcomes.

## 5 Conclusion

This network meta-analysis comprehensively compared the effects of four virtual reality (VR) intervention measures on the cognitive and motor functions of patients with cognitive impairment, providing important reference for clinicians and researchers. Preliminary evidence suggests that VR-SPT may have a positive effect on cognitive outcomes in patients with cognitive impairment, particularly those with MCI. However, this benefit did not robustly extend to specific cognitive domains. The potential advantage for executive function was based on only two studies, should be considered exploratory, and is supported by evidence of very low certainty, thus requiring confirmation in future trials with adequate sample sizes. Concurrently, the existing evidence suggests that VR-CCG+SPT may offer a relative advantage in improving motor function in patients with cognitive impairment, and also exerts positive effects on cognitive function in those with dementia. The selection of a VR intervention should be guided by the participant’s living environment and preferences, in conjunction with the aforementioned preliminary findings, to delay cognitive and motor function decline in patients with cognitive impairment. However, due to limitations such as a relatively weak evidence base for key comparisons, overly wide confidence intervals for some results, and the use of overlapping MoCA domains in different outcome networks, the results of this study should be interpreted with caution, and the above conclusions require further research for validation. Future research should prioritize conducting well-designed, multi-arm randomized controlled trials to provide more direct and reliable evidence concerning the relative efficacy of various VR interventions.

## Data Availability

The original contributions presented in this study are included in this article/Supplementary material, further inquiries can be directed to the corresponding authors.
