# Supplementary material for: Effects of different interventions based on virtual reality on cognitive and motor function in elderly people with mild cognitive impairment or dementia: a systematic review and network meta-analysis
Source: Front Aging Neurosci. 2026 Aug 10;18:1888654. doi: 10.3389/fnagi.2026.1888654 (PMC13500774; doi:10.3389/fnagi.2026.1888654)
Supplement: Supplementary file 1 [file Data_Sheet_1.docx]

Content

[Appendix 1 Search strategy	2](#_Toc1957609583)

[Appendix 2 The measurement tools for different outcomes	25](#_Toc2094789441)

[Appendix 3 The classifications of VR interventions	28](#_Toc1279225969)

[Appendix 4 Study characteristics	29](#_Toc1492070866)

[Appendix 5 The list for the included studies	38](#_Toc1068498843)

[Appendix 6 Risk of bias	49](#_Toc1001798087)

[Appendix 7 The analysis for outcomes	51](#_Toc948655729)

[7.1 Traditional pairwise meta-analysis	51](#_Toc1138241975)

[7.1.1 Global cognition	51](#_Toc648546349)

[7.1.2 Primary analysis for global cognition in MCI group	53](#_Toc1638979118)

[7.1.3 Primary analysis for global cognition in AD group	54](#_Toc549296157)

[7.1.4 Executive function	55](#_Toc2135795893)

[7.1.5 Primary analysis for executive function in MCI group	56](#_Toc1132414046)

[7.1.6 Motor function	57](#_Toc1482791408)

[7.1.7 Primary analysis for motor function in MCI group	58](#_Toc1874954468)

[7. 2 Network analysis	60](#_Toc184707598)

[7.2.1 Global cognition	60](#_Toc1266729671)

[7.2.2 Primary analysis for global cognition in MCI group	62](#_Toc1920187786)

[7.2.3 Primary analysis for global cognition in AD group	64](#_Toc211872186)

[7.2.4 Executive function	66](#_Toc407943376)

[7.2.5 Primary analysis for executive function in MCI group	68](#_Toc1536519208)

[7.2.6 Motor function	71](#_Toc787473681)

[7.2.7 Primary analysis for motor function in MCI group	73](#_Toc128440106)

[Appendix 8 The results of meta regression analysis	77](#_Toc471796307)

[Appendix 9 The distribution of key covariates in each VR intervention group	80](#_Toc970907025)

[Appendix 10 The strength of evidence	83](#_Toc1453619269)

# Appendix 1 Search strategy

Table S1 Search strategy in PubMed

| **Search number** | **Query** | **Results** | **Time** | **Date** |
| --- | --- | --- | --- | --- |
| 1 | "Virtual Reality"[Mesh] | 9,421 | 23:51:44 | 2025/10/24 |
| 2 | "virtual realities"[Title/Abstract] OR "reality virtual"[Title/Abstract] OR "telerehabilitation*"[Title/Abstract] OR "immersive virtual realities"[Title/Abstract] OR "semi immersive virtual realities"[Title/Abstract] OR "non immersive virtual realities"[Title/Abstract] OR "virtual reality technology"[Title/Abstract] OR "video game*"[Title/Abstract] OR "videogame"[Title/Abstract] OR "wii"[Title/Abstract] OR "computer game"[Title/Abstract] | 12,348 | 23:52:37 | 2025/10/24 |
| 3 | #1 OR #2 | 21,108 | 23:53:20 | 2025/10/24 |
| 4 | "Virtual Reality Exposure Therapy"[MeSH Terms] | 1,232 | 23:53:33 | 2025/10/24 |
| 5 | "virtual reality immersion therapy"[Title/Abstract] OR "virtual reality therapy*"[Title/Abstract] OR "reality therapy* virtual"[Title/Abstract] OR "therapy* virtual reality"[Title/Abstract] | 307 | 23:53:42 | 2025/10/24 |
| 6 | #4 OR #5 | 1,448 | 23:59:05 | 2025/10/24 |
| 7 | "Exergaming"[MeSH Terms] | 211 | 23:59:18 | 2025/10/24 |
| 8 | "virtual reality exercise*"[Title/Abstract] OR "exercise* virtual reality"[Title/Abstract] | 132 | 23:59:29 | 2025/10/24 |
| 9 | #7 OR #8 | 330 | 23:59:42 | 2025/10/24 |
| **Search number** | **Query** | **Results** | **Time** | **Date** |
| 10 | "Cognitive Dysfunction"[MeSH Terms] | 50,740 | 23:59:51 | 2025/10/24 |
| 11 | "mild cognitive impairment*"[Title/Abstract] OR "cognitive impairment* mild"[Title/Abstract] OR "impairment* mild cognitive"[Title/Abstract] | 31,521 | 0:00:23 | 2025/10/25 |
| 12 | #10 OR #11 | 66,185 | 0:00:35 | 2025/10/25 |
| 13 | "Dementia"[MeSH Terms] | 232,181 | 0:00:45 | 2025/10/25 |
| 14 | "Dementias"[Title/Abstract] OR "amentia*"[Title/Abstract] OR (("Senile"[All Fields] OR "seniles"[All Fields] OR "senility"[All Fields]) AND "paranoid dementia*"[Title/Abstract]) OR (("dementia"[MeSH Terms] OR "dementia"[All Fields] OR "Dementias"[All Fields] OR "dementia s"[All Fields]) AND "senile paranoid"[Title/Abstract]) OR (("Paranoid"[All Fields] OR "paranoids"[All Fields]) AND "dementia* senile"[Title/Abstract]) | 8,816 | 0:00:54 | 2025/10/25 |
| 15 | #13 OR #14 | 234,921 | 0:01:03 | 2025/10/25 |
| 16 | "Alzheimer Disease"[Mesh] | 136,714 | 0:01:12 | 2025/10/25 |
| 17 | "alzheimer syndrome"[Title/Abstract] OR "alzheimer* diseases"[Title/Abstract] OR "alzheimer dementia*"[Title/Abstract] OR "dementia alzheimer"[Title/Abstract] | 3,603 | 0:01:25 | 2025/10/25 |
| 18 | #16 OR #17 | 138,606 | 0:01:45 | 2025/10/25 |
| 19 | "Cognition"[MeSH Terms] | 221,958 | 0:01:57 | 2025/10/25 |
| 20 | "Cognitions"[Title/Abstract] OR "cognitive function*"[Title/Abstract] OR "function* cognitive"[Title/Abstract] | 113,502 | 0:02:10 | 2025/10/25 |

| **Search number** | **Query** | **Results** | **Time** | **Date** |
| --- | --- | --- | --- | --- |
| 21 | #19 OR #20 | 298,893 | 0:02:22 | 2025/10/25 |
| 22 | "motor function"[Title/Abstract] OR "movement ability"[Title/Abstract] OR "athletic ability"[Title/Abstract] OR "exercise performance"[Title/Abstract] OR "exercise capacity"[Title/Abstract] | 61,405 | 0:02:39 | 2025/10/25 |
| 23 | #3 OR #6 OR #9 | 22,210 | 0:02:56 | 2025/10/25 |
| 24 | #12 OR #15 OR #18 | 278,782 | 0:03:08 | 2025/10/25 |
| 25 | "Randomized Controlled Trials as Topic"[Mesh] | 191,903 | 0:03:59 | 2025/10/25 |
| 26 | "Clinical Trials, Randomized"[Title/Abstract] OR "Trials, Randomized Clinical"[Title/Abstract] | 247 | 0:04:38 | 2025/10/25 |
| 27 | #25 OR #26 | 192,109 | 0:05:02 | 2025/10/25 |
| 28 | (#23 AND #24) AND #27 | 36 | 0:06:23 | 2025/10/25 |
| 29 | #23 AND #24 | 438 | 0:14:08 | 2025/10/25 |

Table S2 Search strategy in Web of Science

| # | Search Query | Database | Results | Date Run |
| --- | --- | --- | --- | --- |
| 1 | Virtual Reality (Topic) OR Virtual Realities (Topic) OR Telerehabilitation* (Topic) OR Immersive VR (Topic) OR Semi-immersive (Topic) OR virtual reality technology (Topic) OR video game* (Topic) OR wii (Topic) OR Virtual Reality Exposure Therapy (Topic) OR Virtual Reality Therapy* (Topic) OR Therapy*, Virtual Reality (Topic) OR Exergaming (Topic) OR Virtual Reality Exercise* (Topic) OR Exercise*, Virtual Reality (Topic) ,Timespan: All years (Publication Date) | Web of Science Core Collection | 118708 | Sat Oct 25 2025 12:34:02 GMT+0800 |
| 2 | mild cognitive impairment (Topic) OR Mild Cognitive Impairment* (Topic) OR Cognitive Impairment*, Mild (Topic) OR Impairment*, Mild Cognitive (Topic) OR Dementia* (Topic) OR Amentia* (Topic) OR Senile Paranoid Dementia* (Topic) OR Paranoid Dementia*, Senile (Topic) OR Familial Dementia (Topic) OR Virtual Reality Therapy* (Topic) OR Dementia, Familial (Topic) OR Frontotemporal Dementia (Topic) OR Frontotemporal Lobe Dementia (Topic) OR Semantic Dementia (Topic) OR Alzheimer Disease (Topic) OR Alzheimer Syndrome (Topic) OR Alzheimer* Diseases (Topic) OR Alzheimer Dementia* (Topic) ,Timespan: All years (Publication Date) | Web of Science Core Collection | 317750 | Sat Oct 25 2025 12:39:40 GMT+0800 |

| # | Search Query | Database | Results | Date Run |
| --- | --- | --- | --- | --- |
| 3 | Cognition (Topic) OR Cognitions (Topic) OR Cognitive Function* (Topic) OR Function*, Cognitive (Topic) ,Timespan: All years (Publication Date) | Web of Science Core Collection | 352043 | Sat Oct 25 2025 12:40:44 GMT+0800 |
| 4 | athletic ability (Topic) OR motor function (Topic) OR athletic ability (Topic) OR exercise performance (Topic) OR exercise Capacity (Topic) ,Timespan: All years (Publication Date) | Web of Science Core Collection | 212800 | Sat Oct 25 2025 12:41:32 GMT+0800 |
| 5 | Randomized Controlled Trials as Topic (Topic) OR Clinical Trials, Randomized (Topic) OR Trials, Randomized Clinical (Topic) ,Timespan: All years (Publication Date) | Web of Science Core Collection | 290524 | Sat Oct 25 2025 12:42:18 GMT+0800 |
| 6 | #1 AND #2 | Web of Science Core Collection | 7904 | Sat Oct 25 2025 12:43:05 GMT+0800 |
| 7 | #1 AND #2 AND #3 | Web of Science Core Collection | 1367 | Sat Oct 25 2025 12:43:46 GMT+0800 |

| # | Search Query | Database | Results | Date Run |
| --- | --- | --- | --- | --- |
| 8 | #1 AND #2 AND #4 | Web of Science Core Collection | 1245 | Sat Oct 25 2025 12:44:40 GMT+0800 |
| 9 | #1 AND #2 AND #5 | Web of Science Core Collection | 868 | Sat Oct 25 2025 12:45:04 GMT+0800 |

Table S3 Search strategy in Embase

| No. | Query | Results | Date |
| --- | --- | --- | --- |
| #1 | 'virtual reality'/exp OR 'virtual reality' OR 'virtual realities':ti,ab,kw OR 'reality virtual':ti,ab,kw OR 'telerehabilitation*':ti,ab,kw OR 'immersive virtual realities':ti,ab,kw OR 'virtual reality technology':ti,ab,kw OR 'video game*':ti,ab,kw OR 'videogame':ti,ab,kw OR 'wii':ti,ab,kw OR 'computer game':ti,ab,kw OR 'virtual reality exposure therapy'/exp OR 'virtual reality exposure therapy' OR 'virtual reality immersion therapy':ti,ab,kw OR 'virtual reality therapy*':ti,ab,kw OR 'reality therapy* virtual':ti,ab,kw OR 'therapy* virtual reality':ti,ab,kw OR 'exergaming'/exp OR 'exergaming' OR 'virtual reality exercise*':ti,ab,kw OR 'exercise* virtual reality':ti,ab,kw | 62905 | 25-Oct-25 |
| #2 | cognitive dysfunction'/exp OR 'mild cognitive impairment*':ti,ab,kw OR 'cognitive impairment* mild':ti,ab,kw OR 'impairment* mild cognitive':ti,ab,kw OR 'amentia*':ti,ab,kw OR 'senile':ti,ab,kw OR 'seniles':ti,ab,kw OR 'senility':ti,ab,kw OR 'paranoid dementia*':ti,ab,kw OR 'dementia'/exp OR 'dementia':ti,ab,kw OR 'dementias':ti,ab,kw OR 'dementia s':ti,ab,kw OR 'senile paranoid':ti,ab,kw OR 'paranoid':ti,ab,kw OR 'paranoids':ti,ab,kw OR 'dementia* senile':ti,ab,kw OR 'alzheimer disease'/exp OR 'alzheimer syndrome':ti,ab,kw OR 'alzheimer* diseases':ti,ab,kw OR 'alzheimer dementia*':ti,ab,kw OR 'dementia alzheimer':ti,ab,kw | 807375 | 25-Oct-25 |
| #3 | 'cognition'/exp OR 'cognitions':ti,ab,kw OR 'cognitive function*':ti,ab,kw OR 'function* cognitive':ti,ab,kw | 3710970 | 25-Oct-25 |
| #4 | 'motor function':ti,ab,kw OR 'movement ability':ti,ab,kw OR 'athletic ability':ti,ab,kw OR 'exercise performance':ti,ab,kw OR 'exercise capacity':ti,ab,kw | 96140 | 25-Oct-25 |
| #5 | 'randomized controlled trials as topic'/exp OR 'clinical trials randomized':ti,ab,kw OR 'trials randomized clinical':ti,ab,kw | 303343 | 25-Oct-25 |
| #6 | #1 AND #2 AND #5 | 158 | 25-Oct-25 |
| No. | Query | Results | Date |
| #7 | #1 AND #2 AND #3 | 2172 | 25-Oct-25 |
| #8 | #1 AND #2 AND #4 | 113 | 25-Oct-25 |
| #9 | #1 AND #2 AND #3 AND #4 AND #5 | 3 | 25-Oct-25 |
| #10 | #1 AND #2 AND #3 AND #5 | 126 | 25-Oct-25 |
| #11 | #1 AND #2 AND #4 AND #5 | 5 | 25-Oct-25 |

Table S4 Search strategy in the Cochrane Library

| Search Name: | LJS |  |
| --- | --- | --- |
| Date Run: | 25/10/2025 16:45:06 |  |
| Comment: | None |  |
| ID | Search Hits | Results |
| #1 | MeSH descriptor: [Virtual Reality] explode all trees | 1586 |
| #2 | (virtual realities):ti,ab,kw OR (reality virtual):ti,ab,kw OR (telerehabilitation*):ti,ab,kw OR (immersive virtual realities):ti,ab,kw OR (virtual reality technology):ti,ab,kw OR (video game*):ti,ab,kw OR (videogame):ti,ab,kw OR (wii):ti,ab,kw OR (computer game):ti,ab,kw OR (virtual reality immersion therapy):ti,ab,kw OR (virtual reality therapy*):ti,ab,kw OR (reality therapy* virtual):ti,ab,kw OR (therapy* virtual reality):ti,ab,kw OR (virtual reality exercise*):ti,ab,kw OR (exercise* virtual reality):ti,ab,kw | 14569 |
| #3 | #1 OR #2 | 14586 |
| #4 | MeSH descriptor: [Cognitive Dysfunction] explode all trees | 4806 |
| #5 | (mild cognitive impairment*):ti,ab,kw OR (cognitive impairment* mild):ti,ab,kw OR (impairment* mild cognitive):ti,ab,kw | 7239 |
| #6 | MeSH descriptor: [Dementia] explode all trees | 10137 |
| #7 | (Dementias):ti,ab,kw OR (amentia*):ti,ab,kw OR (Senile):ti,ab,kw OR (seniles):ti,ab,kw OR (senility):ti,ab,kw OR (paranoid dementia*):ti,ab,kw OR (Dementia):ti,ab,kw OR (Dementias):ti,ab,kw OR (dementia s):ti,ab,kw OR (senile paranoid):ti,ab,kw OR (Paranoid):ti,ab,kw OR (paranoids):ti,ab,kw OR (dementia* senile):ti,ab,kw | 21826 |

| ID | Search Hits | Results |
| --- | --- | --- |
| #8 | #4 OR #5 OR #6 OR #7 | 31135 |
| #9 | #3 AND #8 | 614 |
| #10 | MeSH descriptor: [Cognition] explode all trees | 16801 |
| #11 | (Cognitions):ti,ab,kw OR (cognitive function*):ti,ab,kw OR (function* cognitive):ti,ab,kw | 52906 |
| #12 | #10 OR #11 | 62548 |
| #13 | (motor function):ti,ab,kw OR (movement ability):ti,ab,kw OR (athletic ability):ti,ab,kw OR (exercise performance):ti,ab,kw OR (exercise capacity):ti,ab,kw | 61362 |
| #14 | MeSH descriptor: [Randomized Controlled Trial] explode all trees | 34 |
| #15 | (clinical trials randomized):ti,ab,kw OR (trials randomized clinical):ti,ab,kw | 193779 |
| #16 | #14 OR #15 | 193810 |
| #17 | #9 AND #12 | 370 |
| #18 | #9 AND #13 | 109 |
| #19 | #9 AND #15 | 88 |
| #20 | #9 AND #15 AND #12 | 67 |
| #21 | #9 AND #15 AND #13 | 23 |

Table S5 Search strategy in CINAHL

| S# | Query (user-entered) | Search run Date and Time | Results (count) | Interface | Database(s) |
| --- | --- | --- | --- | --- | --- |
| S1 | MH (MH "Virtual Reality+") OR TI (Virtual Realities OR Telerehabilitation* OR Immersive VR OR Semi immersive VR OR Non immersive VR OR virtual reality technology OR video game* OR videogame OR wii OR computer game) OR AB (Virtual Realities OR Telerehabilitation* OR Immersive VR OR Semi immersive VR OR Non immersive VR OR virtual reality technology OR video game* OR videogame OR wii OR computer game) | 2025-10-25T15:51:15.344Z | 13456 | EBSCOhost Research Databases | CINAHL Complete |
| S2 | MH (MM "Virtual Reality Exposure Therapy") OR TI (Virtual Reality Immersion Therapy* OR Virtual Reality Therapy* OR Reality Therapy* Virtual OR Therapy* Virtual Reality) OR AB (Virtual Reality Immersion Therapy* OR Virtual Reality Therapy* OR Reality Therapy* Virtual OR Therapy* Virtual Reality) | 2025-10-25T15:53:52.788Z | 555 | EBSCOhost Research Databases | CINAHL Complete |
| S3 | MH (MH "Exergames") OR TI (Virtual Reality Exercise* OR Exercise* Virtual Reality) OR AB (Virtual Reality Exercise* OR Exercise* Virtual Reality) | 2025-10-25T15:54:35.527Z | 204 | EBSCOhost Research Databases | CINAHL Complete |
| S4 | S1 OR S2 OR S3 | 2025-10-25T15:55:44.952Z | 13456 | EBSCOhost Research Databases | CINAHL Complete |
| S# | Query (user-entered) | Search run Date and Time | Results (count) | Interface | Database(s) |
| S5 | MH (MH "Mild Cognitive Impairment") OR TI (Cognitive Dysfunction OR Mild Cognitive Impairment* OR Cognitive Impairment* Mild OR Impairment* Mild Cognitive) AND AB (Cognitive Dysfunction OR Mild Cognitive Impairment* OR Cognitive Impairment* Mild OR Impairment* Mild Cognitive) | 2025-10-25T15:59:54.358Z | 4246 | EBSCOhost Research Databases | CINAHL Complete |
| S6 | MH (MH "Dementia+") OR TI (Dementias OR amentia* OR Senile OR seniles OR senility OR paranoid dementia* OR Dementia OR Dementias OR dementia s OR senile paranoid OR Paranoid OR paranoids OR dementia* senile) OR AB (Dementias OR amentia* OR Senile OR seniles OR senility OR paranoid dementia* OR Dementia OR Dementias OR dementia s OR senile paranoid OR Paranoid OR paranoids OR dementia* senile) | 2025-10-25T16:03:59.947Z | 72573 | EBSCOhost Research Databases | CINAHL Complete |
| S7 | MH (MH "Alzheimer's Disease") OR TI (alzheimer syndrome OR alzheimer* diseases OR alzheimer dementia* OR dementia alzheimer) OR AB (alzheimer syndrome OR alzheimer* diseases OR alzheimer dementia* OR dementia alzheimer) | 2025-10-25T16:04:43.406Z | 42011 | EBSCOhost Research Databases | CINAHL Complete |
| S8 | S5 OR S6 OR S7 | 2025-10-25T16:06:27.923Z | 100019 | EBSCOhost Research Databases | CINAHL Complete |

| S# | Query (user-entered) | Search run Date and Time | Results (count) | Interface | Database(s) |
| --- | --- | --- | --- | --- | --- |
| S9 | MH (MH "Athletic Ability") OR TI (motor function OR movement ability OR athletic ability OR exercise performance OR exercise capacity) OR AB (motor function OR movement ability OR athletic ability OR exercise performance OR exercise capacity) | 2025-10-25T16:09:06.467Z | 25147 | EBSCOhost Research Databases | CINAHL Complete |
| S10 | MH (MH "Randomized Controlled Trials+") OR TI (clinical trials randomized OR trials randomized clinical) OR AB (clinical trials randomized OR trials randomized clinical) | 2025-10-25T16:09:45.333Z | 53366 | EBSCOhost Research Databases | CINAHL Complete |
| S11 | S4 AND S8 | 2025-10-25T16:11:12.284Z | 259 | EBSCOhost Research Databases | CINAHL Complete |
| S12 | MH (MH "Cognitions") OR TI (Cognitions OR cognitive function* OR function* cognitive) OR AB (Cognitions OR cognitive function* OR function* cognitive) | 2025-10-25T16:12:22.748Z | 66974 | EBSCOhost Research Databases | CINAHL Complete |
| S13 | S10 AND S11 | 2025-10-25T16:15:22.446Z | 6 | EBSCOhost Research Databases | CINAHL Complete |
| S14 | S9 AND S11 | 2025-10-25T16:18:04.784Z | 6 | EBSCOhost Research Databases | CINAHL Complete |
| S15 | S11 AND S12 | 2025-10-25T16:18:49.327Z | 70 | EBSCOhost Research Databases | CINAHL Complete |
| S16 | S11 AND S9 AND S12 | 2025-10-25T16:19:39.808Z | 6 | EBSCOhost Research Databases | CINAHL Complete |

Table S6 Search strategy in IEEE Xplore

| Set Number | Search Query | Refined By | Number of Results | Date of Search |
| --- | --- | --- | --- | --- |
| 1 | ("All Metadata":Virtual Reality*) OR ("All Metadata":Reality, Virtual ) OR ("All Metadata":Telerehabilitation* ) OR ("All Metadata":Immersive VR) OR ("All Metadata":Semi-immersive VR) OR ("All Metadata":Non-immersive VR) OR ("All Metadata":virtual reality technology) OR ("All Metadata":video game*) OR ("All Metadata":wii) OR ("All Metadata":computer game) | Search Latest Date: 10/25/1884-10/25/2025 | 107484 | 25-Oct-25 |
| 2 | ("All Metadata":Virtual Reality Exposure Therapy) OR ("All Metadata":Virtual Reality Therapy* ) OR ("All Metadata":Exergaming) OR ("All Metadata":Virtual Reality Exercise*) | Search Latest Date: 10/25/1884-10/25/2025 | 897 | 25-Oct-25 |
| 3 | (((All Metadata:Virtual Reality*) OR (All Metadata:Reality, Virtual ) OR (All Metadata:Telerehabilitation* ) OR (All Metadata:Immersive VR) OR (All Metadata:Semi-immersive VR) OR (All Metadata:Non-immersive VR) OR (All Metadata:virtual reality technology) OR (All Metadata:video game*) OR (All Metadata:wii) OR (All Metadata:computer game) refined by:Search Latest Date:10/25/1884-10/25/2025 )) OR ((All Metadata:Virtual Reality Exposure Therapy) OR (All Metadata:Virtual Reality Therapy* ) OR (All Metadata:Exergaming) OR (All Metadata:Virtual Reality Exercise*) refined by:Search Latest Date:10/25/1884-10/25/2025 ) | Search Latest Date: 10/25/1884-10/25/2025 | 107504 | 25-Oct-25 |
| 4 | ("All Metadata":Cognitive Dysfunction) OR ("All Metadata":Mild Cognitive Impairment*) OR ("All Metadata":Impairment*, Mild Cognitive) | Search Latest Date: 10/25/1884-10/25/2025 | 3977 | 25-Oct-25 |
| Set Number | Search Query | Refined By | Number of Results | Date of Search |
| 5 | ("All Metadata":dementia*) OR ("All Metadata":Amentia*) OR ("All Metadata":Senile Paranoid Dementia* ) OR ("All Metadata":Familial Dementia) OR ("All Metadata":Frontotemporal Dementia) OR ("All Metadata":Frontotemporal Lobe Dementia) OR ("All Metadata":Semantic Dementia) OR ("All Metadata":Alzheimer Disease) OR ("All Metadata":Alzheimer Syndrome) OR ("All Metadata":Dementia, Alzheimer) OR ("All Metadata":Alzheimer Dementia* ) | Search Latest Date: 10/25/1884-10/25/2025 | 9172 | 25-Oct-25 |
| 6 | ("All Metadata":Cognition*) OR ("All Metadata":Cognitive Function* ) OR ("All Metadata":Function*, Cognitive) | Search Latest Date: 10/25/1884-10/25/2025 | 82797 | 25-Oct-25 |
| 7 | ("All Metadata":athletic ability) OR ("All Metadata":motor function) OR ("All Metadata":athletic ability) OR ("All Metadata":exercise performance) OR ("All Metadata":exercise Capacity) | Search Latest Date: 10/25/1884-10/25/2025 | 75711 | 25-Oct-25 |
| 8 | (((All Metadata:Cognitive Dysfunction) OR (All Metadata:Mild Cognitive Impairment*) OR (All Metadata:Impairment*, Mild Cognitive))) OR ((All Metadata:dementia*) OR (All Metadata:Amentia*) OR (All Metadata:Senile Paranoid Dementia* ) OR (All Metadata:Familial Dementia) OR (All Metadata:Frontotemporal Dementia) OR (All Metadata:Frontotemporal Lobe Dementia) OR (All Metadata:Semantic Dementia) OR (All Metadata:Alzheimer Disease) OR (All Metadata:Alzheimer Syndrome) OR (All Metadata:Dementia, Alzheimer) OR (All Metadata:Alzheimer Dementia* ) refined by:Search Latest Date:10/25/1884-10/25/2025 ) | Search Latest Date: 10/25/1884-10/25/2025 | 10279 | 25-Oct-25 |
| Set Number | Search Query | Refined By | Number of Results | Date of Search |
| 9 | (((((All Metadata:Virtual Reality*) OR (All Metadata:Reality, Virtual ) OR (All Metadata:Telerehabilitation* ) OR (All Metadata:Immersive VR) OR (All Metadata:Semi-immersive VR) OR (All Metadata:Non-immersive VR) OR (All Metadata:virtual reality technology) OR (All Metadata:video game*) OR (All Metadata:wii) OR (All Metadata:computer game) refined by:Search Latest Date:10/25/1884-10/25/2025 )) AND ((No Keywords Specified))) AND ((All Metadata:Cognitive Dysfunction) OR (All Metadata:Mild Cognitive Impairment*) OR (All Metadata:Impairment*, Mild Cognitive))) OR ((All Metadata:dementia*) OR (All Metadata:Amentia*) OR (All Metadata:Senile Paranoid Dementia* ) OR (All Metadata:Familial Dementia) OR (All Metadata:Frontotemporal Dementia) OR (All Metadata:Frontotemporal Lobe Dementia) OR (All Metadata:Semantic Dementia) OR (All Metadata:Alzheimer Disease) OR (All Metadata:Alzheimer Syndrome) OR (All Metadata:Dementia, Alzheimer) OR (All Metadata:Alzheimer Dementia* ) refined by:Search Latest Date:10/25/1884-10/25/2025 ) | Search Latest Date: 10/25/1884-10/25/2025 | 9297 | 25-Oct-25 |
| 10 | ("All Metadata":Randomized Controlled Trials as Topic) OR ("All Metadata":Clinical Trials, Randomized) | Search Latest Date: 10/25/1884-10/25/2025 | 613 | 25-Oct-25 |

| Set Number | Search Query | Refined By | Number of Results | Date of Search |
| --- | --- | --- | --- | --- |
| 11  (#9 AND #10 ) | (((((((No Keywords Specified))) AND ((All Metadata:Virtual Reality*) OR (All Metadata:Reality, Virtual ) OR (All Metadata:Telerehabilitation* ) OR (All Metadata:Immersive VR) OR (All Metadata:Semi-immersive VR) OR (All Metadata:Non-immersive VR) OR (All Metadata:virtual reality technology) OR (All Metadata:video game*) OR (All Metadata:wii) OR (All Metadata:computer game) refined by:Search Latest Date:10/25/1884-10/25/2025 )) AND ((No Keywords Specified))) AND ((All Metadata:Cognitive Dysfunction) OR (All Metadata:Mild Cognitive Impairment*) OR (All Metadata:Impairment*, Mild Cognitive))) OR ((All Metadata:dementia*) OR (All Metadata:Amentia*) OR (All Metadata:Senile Paranoid Dementia* ) OR (All Metadata:Familial Dementia) OR (All Metadata:Frontotemporal Dementia) OR (All Metadata:Frontotemporal Lobe Dementia) OR (All Metadata:Semantic Dementia) OR (All Metadata:Alzheimer Disease) OR (All Metadata:Alzheimer Syndrome) OR (All Metadata:Dementia, Alzheimer) OR (All Metadata:Alzheimer Dementia* ) refined by:Search Latest Date:10/25/1884-10/25/2025 )) AND ((All Metadata:Randomized Controlled Trials as Topic) OR (All Metadata:Clinical Trials, Randomized) refined by:Year:1884-2026 ) | Search Latest Date: 10/25/1884-10/25/2025 | 19 | 25-Oct-25 |

| Set Number | Search Query | Refined By | Number of Results | Date of Search |
| --- | --- | --- | --- | --- |
| 12  (#9 AND #6) | (((("All Metadata":"Virtual Reality*") OR ("All Metadata":"Reality, Virtual") OR ("All Metadata":"Immersive VR") OR ("All Metadata":"Semi-immersive VR") OR ("All Metadata":"virtual reality technology") OR ("All Metadata":"wii") OR ("All Metadata":"computer game")) AND ((("All Metadata":"Cognitive Dysfunction") OR ("All Metadata":"Mild Cognitive Impairment*") OR ("All Metadata":"dementia*") OR ("All Metadata":"Amentia*") OR ("All Metadata":"Familial Dementia") OR ("All Metadata":"Frontotemporal Lobe Dementia") OR ("All Metadata":"Semantic Dementia") OR ("All Metadata":"Alzheimer Disease") OR ("All Metadata":"Alzheimer Syndrome") OR ("All Metadata":"Dementia, Alzheimer") OR ("All Metadata":"Alzheimer Dementia*"))) AND ((("All Metadata":"Cognition*") OR ("All Metadata":"Cognitive Function*") OR ("All Metadata":"Function*, Cognitive"))))) | Search Latest Date: 10/25/1884-10/25/2025 | 76 | 26-Oct-25 |

| Set Number | Search Query | Refined By | Number of Results | Date of Search |
| --- | --- | --- | --- | --- |
| 13  (#9 AND #7) | ((("All Metadata":"Virtual Reality*") OR ("All Metadata":"Reality, Virtual") OR ("All Metadata":"Immersive VR") OR ("All Metadata":"Semi-immersive VR") OR ("All Metadata":"virtual reality technology") OR ("All Metadata":"wii") OR ("All Metadata":"computer game")) AND ((("All Metadata":"Cognitive Dysfunction") OR ("All Metadata":"Mild Cognitive Impairment*") OR ("All Metadata":"dementia*") OR ("All Metadata":"Amentia*") OR ("All Metadata":"Familial Dementia") OR ("All Metadata":"Frontotemporal Lobe Dementia") OR ("All Metadata":"Semantic Dementia") OR ("All Metadata":"Alzheimer Disease") OR ("All Metadata":"Alzheimer Syndrome") OR ("All Metadata":"Dementia, Alzheimer") OR ("All Metadata":"Alzheimer Dementia*"))) AND ((("All Metadata":athletic ability) OR ("All Metadata":motor function) OR ("All Metadata":athletic ability) OR ("All Metadata":exercise performance) OR ("All Metadata":exercise Capacity)))) | Search Latest Date: 10/25/1884-10/25/2025 | 22 | 27-Oct-25 |

Table S7 Search strategy in CNKI

| 序号 | 检索条件 | 检索范围 | 检索时间 | 文献数量 |
| --- | --- | --- | --- | --- |
| 1 | （主题：虚拟现实技术 + 远程康复* + 沉浸式VR + 半沉浸式VR + 非沉浸式VR + 计算机游戏）OR（主题：虚拟现实沉浸疗法 + 虚拟现实疗法 + 虚拟现实锻炼 + 虚拟现实运动） | 资源范围：总库; 中英文扩展; 时间范围：更新时间：不限; | 2025/10/26 9:40 | 21120 |
| 2 | （主题：轻度认知障碍 + 认知障碍 + 轻度神经认知障碍 + 认知能力下降 + MCI）OR（主题：痴呆症 + 老年性偏执型痴呆 + 痴呆 + 老年性妄想性痴呆 + 老年性偏执型痴呆 + 家族性痴呆 + 额颞叶痴呆 + 语义痴呆）OR（主题：阿尔茨海默病综合征 + 阿尔茨海默病） | 资源范围：总库; 中英文扩展; 时间范围：更新时间：不限; | 2025/10/26 9:41 | 117294 |
| 3 | 1 AND 2 | 资源范围：总库; 中英文扩展; 时间范围：更新时间：不限; | 2025/10/26 9:43 | 136 |
| 4 | （主题：认知功能 + 认知能力） | 资源范围：总库; 中英文扩展; 时间范围：更新时间：不限; | 2025/10/26 9:50 | 115378 |
| 5 | （主题：运动功能 + 运动能力） | 资源范围：总库; 中英文扩展; 时间范围：更新时间：不限; | 2025/10/26 9:50 | 76169 |
| 6 | （主题：随机对照 + 随机对照试验 + 随机对照研究 + '随机对照试验(rct)'） | 资源范围：总库; 中英文扩展; 时间范围：更新时间：不限; | 2025/10/26 9:52 | 55742 |

| 序号 | 检索条件 | 检索范围 | 检索时间 | 文献数量 |
| --- | --- | --- | --- | --- |
| 7 | 1 AND 2 AND 4 | 资源范围：总库; 中英文扩展; 时间范围：更新时间：不限; | 2025/10/26 9:54 | 95 |
| 8 | 1 AND 2 AND 5 | 资源范围：总库; 中英文扩展; 时间范围：更新时间：不限; | 2025/10/26 9:59 | 14 |
| 9 | 1 AND 2 AND 5 | 资源范围：总库; 中英文扩展; 时间范围：更新时间：不限; | 2025/10/26 10:03 | 3 |

Table S8 Search strategy in VIP

| 编号 | 检索表达式 | 检索结果 | 检索时间 |
| --- | --- | --- | --- |
| 1# | (((((((题名或关键词=虚拟现实技术 AND 题名或关键词=远程康复)) OR 题名或关键词=沉浸式VR) OR 题名或关键词=半沉浸式VR) OR 题名或关键词=非沉浸式VR) OR (题名或关键词=计算机游戏 OR 题名或关键词=虚拟现实沉浸疗法)) OR ((题名或关键词=虚拟现实疗法 OR 题名或关键词=虚拟现实锻炼) OR 题名或关键词=虚拟现实运动)) | 1135 | 2025/10/26 |
| 2# | ((((((题名或关键词=轻度认知障碍 OR 题名或关键词=认知障碍) OR 题名或关键词=轻度神经认知障碍) OR 题名或关键词=认知能力下降) OR 题名或关键词=MCI) OR (((((((题名或关键词=痴呆症 OR 题名或关键词=老年性偏执型痴呆) OR 题名或关键词=痴呆) OR 题名或关键词=老年性妄想性痴呆) OR 题名或关键词=老年性偏执型痴呆) OR 题名或关键词=家族性痴呆) OR 题名或关键词=额颞叶痴呆) OR 题名或关键词=语义痴呆)) OR (题名或关键词=阿尔茨海默病综合征 OR 题名或关键词=阿尔茨海默病)) | 69118 | 2025/10/26 |
| 3# | (题名或关键词=认知功能 OR 题名或关键词=认知能力) | 56726 | 2025/10/26 |
| 4# | (题名或关键词=运动功能 OR 题名或关键词=运动能力) | 40193 | 2025/10/26 |
| 5# | (题名或关键词=随机对照试验 OR 题名或关键词=RCT) | 15375 | 2025/10/26 |
| 6# | 1# AND 2# AND 3# | 6 | 2025/10/26 |
| 7# | 1# AND 2# AND 4# | 2 | 2025/10/26 |
| 8# | 1# AND 2# AND 5# | 0 | 2025/10/26 |

Table S9 Search strategy in Wanfang

| 序号 | 文献类型 | 检索式 | 检索结果 | 检索时间 |
| --- | --- | --- | --- | --- |
| #1 | 期刊、学位、会议 | 主题:(虚拟现实技术 or 远程康复* or 沉浸式VR or 半沉浸式VR or 非沉浸式VR or 计算机游戏 or 虚拟现实沉浸疗法 or 虚拟现实疗法 or 虚拟现实锻炼 or 虚拟现实运动) | 76173 | 2025-10-26 |
| #2 | 期刊、学位、会议 | 主题:(轻度认知障碍 or 认知障碍 or 轻度神经认知障碍 or 认知能力下降 or MCI) or 主题:(痴呆症 or 老年性偏执型痴呆 or 痴呆 or 老年性妄想性痴呆 or 老年性偏执型痴呆 or 家族性痴呆 or 额颞叶痴呆 or 语义痴呆) or 主题:(阿尔茨海默病综合征 or 阿尔茨海默病) | 290941 | 2025-10-26 |
| #3 | 期刊、学位、会议 | 主题:(认知功能 or 认知能力) | 452241 | 2025-10-26 |
| #4 | 期刊、学位、会议 | 主题:(运动功能 or 运动能力) | 534771 | 2025-10-26 |
| #5 | 期刊、学位、会议 | 主题:(随机对照试验) | 157288 | 2025-10-26 |
| #6 | 期刊、学位、会议 | #1 AND #2 AND #3 | 113 | 2025-10-26 |
| #7 | 期刊、学位、会议 | #1 AND #2 AND #4 | 38 | 2025-10-26 |
| #8 | 期刊、学位、会议 | #1 AND #2 AND #5 | 16 | 2025-10-26 |

# Appendix 2 The measurement tools for different outcomes

Table S10 The measurement tools for different outcomes

| Primary outcomes | Scale name | Relationship between the total score and the effectiveness |
| --- | --- | --- |
| Global cognition function | 1.Mini-Mental State Examination (MMSE)2.The Montreal cognitive assessment (MoCA)3.Clock Drawing Test (CDT) | Higher=Better  No transformation |
| Executive function | 1.The Montreal cognitive assessment (MoCA)2.Phonemic Verbal Fluency Test (VFT-phonemic)3.Semantic Verbal Fluency Test (VFT-semantic)4.Frontal Assessment Battery (FAB)5.Digit Span Test - Backward Span (DST-Backward ) | Higher=Better  No transformation |
| Secondary outcomes | Scale name | Relationship between the total score and the effectiveness |
| Motor function | 1. 2.44-meter Walk Test (2.44m-walk)2. 5-Times Sit-to-Stand Test (5STS)3. 8-foot Up-and-Go Test (8-feet Up and Go)4. Timed Up and Go Test (TUG)5. Instrumented Timed Up and Go Test (iTUG)6. Gait Speed（GS） | Lower=Better  effect size symbol inversion |

Table S11 The usage of various research assessment tools

The decision rule for MoCA assignment: MoCA total score used for global cognition only; MoCA executive subscores, when reported separately, used for executive function.

| ID | study | Global cognition | Executive cognition | Motor function |
| --- | --- | --- | --- | --- |
| 1 | Amjad et al.,2019 | MMSE | MoCA executive subscores |  |
| 2 | Arshad et al.,2023 | MMSE | MoCA executive subscores |  |
| 3 | Buele et al.,2024 | MoCA | MoCA executive subscores |  |
| 4 | Choi et al.,2019 | MoCA | MoCA executive subscores | TUG |
| 5 | Delbroek et al.,2017 | MoCA | MoCA executive subscores | iTUG |
| 6 | Ding,2023 | MMSE | MoCA executive subscores |  |
| 7 | Goumopoulos  et al.,2023 | MoCA | MoCA executive subscores |  |
| 8 | Hagovská et al.,2015 | MMSE |  | TUG |
| 9 | Hsieh et al.,2018 |  |  | TUG |
| 10 | Hu et al.,2018 | MoCA | MoCA executive subscores |  |
| 11 | Hughes et al.,2014 |  |  | GS |
| 12 | Ip et al.,2024 | MoCA | MoCA executive subscores | TUG |
| 13 | Kang et al.,2021 | MMSE |  |  |
| 14 | Li et al.,2023a | MMSE | MoCA executive subscores |  |
| 15 | Liao et al.,2019 |  |  |  |
| 16 | Liao et al.,2020 | MoCA | MoCA executive subscores |  |
| 17 | Li et al.,2021 | MMSE | MoCA executive subscores |  |
| 18 | Li et al.,2023b | MoCA | MoCA executive subscores |  |
| 19 | Lim et al.,2023 | MMSE | MoCA executive subscores |  |
| 20 | Liu et al.,2022 | MoCA | MoCA executive subscores |  |

| ID | study | Global cognition | Executive cognition | Motor function |
| --- | --- | --- | --- | --- |
| 21 | Man et al.,2011 | MMSE |  |  |
| 22 | Manenti et al.,2020 | CDT | VFT-phonemic |  |
| 23 | Oliveira et al.,2021 | MMSE | FAB |  |
| 24 | Padala et al.,2012 | MMSE |  | TUG |
| 25 | Park et al.,2020a | MMSE | DST-Backward |  |
| 26 | Park et al.,2020b | MoCA | MoCA executive subscores |  |
| 27 | Schwenk et al.,2016 | MoCA | MoCA executive subscores | GS |
| 28 | Song,2018 | MMSE | MoCA executive subscores |  |
| 29 | Sun et al.,2021a | MMSE |  | TUG |
| 30 | Sun et al.,2021b | MoCA | MoCA executive subscores | 5STS |
| 31 | Tarnanas et al.,2014 | MMSE | DST-Backward |  |
| 32 | Thapa et al.,2020 | MMSE |  | 8-feet Up and go |
| 33 | Tominari et al.,2021 | MMSE |  |  |
| 34 | Ugur et al.,2020 | MMSE |  | GS |
| 35 | Wang et al.,2021 | MMSE | MoCA executive subscores |  |
| 36 | Xie et al.,2020 | MMSE |  |  |
| 37 | Xue et al.,2021 | MoCA | MoCA executive subscores |  |
| 38 | Xu et al.,2020 | MMSE |  |  |
| 39 | Yang et al.,2019 | MMSE | MoCA executive subscores |  |
| 40 | Yang et al.,2022 | MMSE |  |  |
| 41 | Zheng et al.,2021 | MMSE |  |  |
| 42 | Zheng et al.,2024 | MMSE |  |  |

*Note:* ①MMSE: Mini-Mental State Examination；②MoCA: Montreal Cognitive Assessment；③DST-Backward: Digit Span Test - Backward；④DST-Forward: Digit Span Test - Forward；⑤FAB: Frontal Assessment Battery；⑥VFT: Verbal Fluency Test；⑦DSST: Digit Symbol Substitution Test；⑧TMT-A: Trail Making Test Part A；⑨TMT-B: Trail Making Test Part B；⑩TUG: Timed Up and Go Test；⑪iTUG: Instrumented Timed Up and Go Test；⑫GS: Gait Speed Test；⑬6mwt: 6-Minute Walk Test；⑭BBS: Berg Balance Scale；⑮5STS: Five Times Sit-to-Stand Test；⑯8-Foot Up and Go: 8-Foot Up and Go Test (=2.44m Walk)；⑰SPPB: Short Physical Performance Battery (Containing TST sub-items)；⑱TST: Tandem Stand Test (Belonging to SPPB)；⑲SDST: Symbol Digit Substitution Test；⑳CDT: Clock Drawing Test;

# Appendix 3 The classifications of VR interventions

To compare the effects of different types of VR interventions, we classified them based on the degree of VR immersion and the specific intervention content as follows: (1) VR Comprehensive Cognitive Game (VR-CCG): This intervention integrates virtual reality technology with one or more game genres to deliver comprehensive cognitive training. The training tasks are designed to engage multiple cognitive domains, including memory, attention, language, visuospatial abilities, executive functions, and processing speed. Specific training content encompasses a range of activities such as VR supermarket shopping, virtual kitchen tasks, organizing items in a virtual office, graphic and number matching, navigation-based flight training, and rhythm-based games, all implemented within immersive virtual environments.

(2) VR Comprehensive Cognitive Game + Sport Training (VR-CCG + SPT): This intervention combines cognitive game-based training with physical exercise in a virtual reality environment. The physical training component incorporates modalities such as aerobic exercise, strength training, balance and gait training, yoga, and stretching.
(3) VR Sport Training (VR-SPT): This intervention involves the implementation of structured physical exercise within a virtual reality environment. The exercises include modalities such as aerobic, strength, balance, and gait training, as well as yoga and stretching routines.
(4) VR Reminiscence Therapy (VR-MT): This intervention leverages VR technology to create immersive environments designed to evoke the recall of autobiographical memories and past experiences.

# Appendix 4 Study characteristics

Table S12 Study characteristics

| Study | Region | Study type | Participants | | | | Intervention | Interventions Classification | The degree of VR immersion | Interactive device | Duration, Frequency  and cycle | Control group classification | Control | Outcomes |
| --- | --- | --- | --- | --- | --- | --- | --- | --- | --- | --- | --- | --- | --- | --- |
|  |  |  | Dementia/MCI | Living | Mean age  (Mean ± SD) | Sample size (Male/Female) |  |  |  |  |  |  |  |  |
| Amjad et al.,2019 | China | RCT | MCI | Hospital | NA | IG:22  CG:22 | Xbox 360 Kinect cognitive games | VR Comprehensive Cognitive Game | semi-immersion | Xbox 360 Kinect sensor, game console, display screen | 25-30min, 5times/wk, 6wk | Active control group | Upper limb and lower limb joint movements and stretching exercises | ①；②；⑧；⑨ |
| Arshad et al.,2023 | Pakistan | RCT | MCI | Hospital | IG:62.85±5.56 CG:63.24±5.12 | IG:26(16/10) CG:25(15/10) | Xbox 360 Kinect cognitive games | VR Comprehensive Cognitive Game | semi-immersion | Xbox 360 Kinect sensor, game console, display screen | 30min, 5times/wk, 6wk | Active control group | Regular exercise training | ①；②；⑥；⑧；⑨ |
| Buele et al.,2024 | Ecuador | RCT | MCI | Community and daycare centers | IG:75.41±5.76 CG:77.35±6.75 | IG:17(7/10) CG:17(4/13) | Dual intervention of exercise and cognitive training based on VR | VR comprehensive cognitive games + Sport training | Full immersion | Head-mounted display, handle | 40min, 2times/wk, 6wk | Active control group | Regular exercise training | ② |
| Choi et al.,2019 | Korea | RCT | MCI | Welfare center | IG:77.27±4.37 CG:75.37±3.97 | IG:30(5/25) CG:30(4/26) | Virtual Kayak Paddling Exercise | VR comprehensive cognitive games + Sport training | semi-immersion | Soft Balancing Bubble, Screen Playing Recorded Video, Oars | 60min, 2times/wk, 6wk | Active control group | Home exercises | ②；⑩；⑭；㉑；㉒ |

| Study | | | Participants | | | | Intervention | | | | | Control group | | Outcomes |
| --- | --- | --- | --- | --- | --- | --- | --- | --- | --- | --- | --- | --- | --- | --- |
| Delbroek et al.,2017 | Belgium | RCT | MCI | Hospital | IG:86.9±5.6 CG:87.5±6.6 | IG:10(2/8) CG:10(5/5) | Cognitive-Motor Dual-Task Training | VR comprehensive cognitive games + Sport training | semi-immersion | Flat panel TV, pressure sensor, wear inertial measurement device | 18-30min, 2times/wk, 6wk | Control group | Usual care | ②；⑪；㉕ |
| Ding,2023 | China | RCT | MCI | Nursing institution | IG:82.81±4.39 CG:82.95±5.64 | IG:21(8/13) CG:21(10/11) | Virtual supermarket cognitive training | VR Comprehensive Cognitive Game | non-immersion | Computer, mouse, keyboard | 30min, 2times/wk, 6wk | Control group | No intervention | ①；② |
| Goumopoulos et al.,2023 | Greece | RCT | MCI | Senior care institutions and communities | IG:74.0±4.5 CG:72.0±3.8 | IG:11(6/5) CG:10(4/6) | Cognitive training in multiple fields and modes based on the COGNIPLAT game platform | VR Comprehensive Cognitive Game | non-immersion | Tablet pc | 60min, 2times/wk, 12wk | Control group | Usual care | ②；③；④；⑧；⑨ |
| Hagovská et al.,2015 | Slovak Republic | RCT | MCI | Hospital | IG:68±4.4 CG:65.9±6.2 | IG:40(22/18) CG:40(19/21) | CogniPlus cognitive training based on VR combined with balance training | VR comprehensive cognitive games + Sport training | non-immersion | Computer | 30min, 2times/wk, 10wk | Active control group | Balance training | ①；⑩；㉔；㉕ |
| Hsieh et al.,2018 | Taiwan | RCT | MCI | Daycare center and medical clinic | IG:76.4±7.6 CG:80.0±7.5 | IG:31(7/24) CG:29(10/19) | VR Tai Chi Training (VR-TC) | VR Sport training | semi-immersion | Xbox 360 Kinect console, display | 60min, 2times/wk, 24wk | Control group | Regular daily activities | ⑩；⑫；⑬ |

| Study | | | Participants | | | | Intervention | | | | | Control group | | Outcomes |
| --- | --- | --- | --- | --- | --- | --- | --- | --- | --- | --- | --- | --- | --- | --- |
| Hu et al.,2018 | China | RCT | MCI | Hospital | IG:73.6±6.3 CG:74.6±5.3 | IG:30(23/7) CG:30(24/6) | Rehabilitation training based on VR-BioMaster virtual scenarios | VR comprehensive cognitive games + Sport training | semi-immersion | Large screen, fixed cyclist | 30min, 5times/wk, 12wk | Control group | Usual care | ②；⑬ |
| Hughes et al.,2014 | USA | RCT | MCI | Community | IG:78.5±7.1 CG:76.2±4.3 | IG:10(2/8) CG:10(4/6) | Interactive video games based on the Nintendo Wii game console | VR comprehensive cognitive games + Sport training | semi-immersion | wii, motion tracker | 90min, 1times/wk, 24wk | Control group | Health education | ⑫ |
| Ip et al.,2024 | Australia | RCT | MCI | Senior care facility | IG:73.67±6.144 CG:80.00±4.359 | IG:9(0/9) CG:9(2/7) | VR Game training | VR comprehensive cognitive games + Sport training | Full immersion | VR & AR platform 3D glasses, motion tracker | 45min, 2times/wk, 8wk | Active control group | Traditional Baduanjin exercise | ②；⑧；⑨；⑩；⑬；⑭ |
| Kang et al.,2021 | Korea | RCT | MCI | Hospital | IG:75.48±4.67 CG:73.28±6.96 | IG:23(6/17) CG:18(6/12) | Multidisciplinary virtual reality cognitive training | VR Comprehensive Cognitive Game | Full immersion | Head-mounted display, hand-held controller | 20-30min, 2times/wk, 4wk | Control group | Usual care | ① |
| Li et al.,2023a | China | RCT | MCI；Dementia | Communities, hospitals and nursing homes | IG:70.97±5.08 CG:70.40±4.52 | IG:30(14/16) CG:30(13/17) | VR Cognitive-motor training | VR comprehensive cognitive games + Sport training | Full immersion | Head-mounted display, hand-held controller | 60min, 1times/wk, 12wk | Control group | Usual care | ①；② |
| Liao et al.,2019 | Taiwan | RCT | MCI | Community and daycare centers | IG:75.5±5.2 CG:73.1±6.8 | IG:18(7/11) CG:16(4/12) | VR Body-Cognitive Training | VR comprehensive cognitive games + Sport training | Full immersion | Head-mounted display, motion controller | 60min, 3times/wk, 12wk | Active control group | Traditional physical and cognitive training combined | ⑧；⑨；㉙ |

| Study | | | Participants | | | | Intervention | | | | | Control group | | Outcomes |
| --- | --- | --- | --- | --- | --- | --- | --- | --- | --- | --- | --- | --- | --- | --- |
| Liao et al.,2020 | Taiwan | RCT | MCI | Community and daycare centers | IG:75.5±5.2 CG:73.1±6.8 | IG:18(7/11) CG:16(4/12) | VR Body-Cognition Training (VRCT) | VR comprehensive cognitive games + Sport training | Full immersion | Head-mounted display, motion controller | 60min, 3times/wk, 12wk | Active control group | Traditional physical and cognitive training combined | ②；㉖；㉗ |
| Li et al.,2021 | China | RCT | MCI | Community | IG:70.63±6.32 CG:71.62±5.27 | IG:30(18/12) CG:29(16/13) | Computer Cognitive Training, CCT | Active control group | non-immersion | Computer | 30min, 5times/wk, 5wk | Control group | No intervention | ①；② |
| Li et al.,2023b | China | RCT | MCI | Nursing institution | IG:83.36±6.27 CG:83.46±8.39 | IG:25(6/19) CG:26(10/16) | Platnoid games based on serious games and dual-task training through aerobic exercises | VR comprehensive cognitive games + Sport training | non-immersion | Tablet pc | 54min, 2times/wk, 12wk | Control group | Usual care | ② |
| Lim et al.,2023 | Korea | RCT | MCI | Hospital | IG:75.42±5.74 CG:73.33±17.52 | IG:12(3/9) CG:12(4/8) | Tablet games based on serious games | VR Comprehensive Cognitive Game | non-immersion | Tablet pc | 30min, 3times/wk, 4wk | Control group | Regular daily activities | ①；②；⑨ |
| Liu et al.,2022 | Taiwan | RCT | MCI | Community | IG:74.6±6.1 CG:73.4±6.5 | IG:16(4/12) CG:17(6/11) | VR Tai Chi Sensory Game Training | VR Sport training | semi-immersion | Kinect sensor, console, display | 50min, 3times/wk, 12wk | Active control group | Traditional Tai Chi training | ②；⑧；⑨； |
| Man et al.,2011 | Hong Kong | RCT | MCI | Community | IG:80.3±1.21 CG:80.28.2±1.31 | IG:20(3/17) CG:24(2/22) | VR Memory training program | VR Comprehensive Cognitive Game | non-immersion | Computer, joystick, keyboard | 30min, 2-3times/wk, 4-5wk | Active control group | Memory training | ① |
| Manenti et al.,2020 | Italy | RCT | MCI | Community | IG:75.3±3.3 CG:78.1±4.1 | IG:18(13/5) CG:17(7/10) | Face-to-face cognitive virtual reality rehabilitation system (VRRS) + remote rehabilitation via home VRRS | VR Comprehensive Cognitive Game | non-immersion | Tablet pc | 60min, 3times/wk, 12wk | Active control group | Regular cognitive training | ⑥；⑧；⑨；⑳；㉚ |

| Study | | | Participants | | | | Intervention | | | | | Control group | | Outcomes |
| --- | --- | --- | --- | --- | --- | --- | --- | --- | --- | --- | --- | --- | --- | --- |
| Oliveira et al.,2021 | Por⑩al | RCT | Dementia | Nursing institution | IG:82.60±5.42 CG:84.14±6.30 | IG:10(3/7) CG:7(2/5) | SLB-VR Cognitive stimulation training | VR Comprehensive Cognitive Game | non-immersion | Laptop screen | 45min, 2times/wk, 8wk | Control group | Usual care | ①；⑤ |
| Padala et al.,2012 | USA | RCT | Dementia | Nursing institution | IG:79.3±9.8 CG:81.6±5.2 | IG:11(3/8) CG:11(3/8) | Balance and strength training based on the Wii-Fit game console | VR Sport training | semi-immersion | Nintendo Wii-Fit game console, connected to mobile TV unit, Wii balance board | 30min, 5times/wk, 8wk | Active control group | Regular exercise training | ①；⑩；⑭；㉓ |
| Park et al.,2020a | Korea | RCT | MCI | Hospital | IG:71.80±6.61 CG:69.45±7.45 | IG:10(3/7) CG:11(4/7) | VR training program based on Korean traditional culture | VR Comprehensive Cognitive Game | Full immersion | HTC Vive head-mounted display, including headphones, two controllers and two infrared laser emitters | 30min, 2times/wk, 12wk | Control group | No intervention | ①；③；④ |
| Park et al.,2020b | Korea | RCT | MCI | Community | IG:75.8±8.5 CG:77.2±7.2 | IG:18(10/8) CG:17(7/10) | Cognitive-motor rehabilitation training based on the MOTOcog-VR system | VR Comprehensive Cognitive Game | semi-immersion | Touch screen displays, grip balloons, various joysticks or accessories | 30min, 5times/wk, 6wk | Active control group | Regular cognitive training | ②；③；④；⑧；⑨ |
| Schwenk et al.,2016 | USA | RCT | MCI | Community Clinic | IG:77.8±6.91 CG:79.0±10.4 | IG:12(5/7) CG:10(5/5) | VR sensor balance training | VR comprehensive cognitive games + Sport training | non-immersion | Computer screen, interactive virtual user interface, inertial sensor | 45min, 2times/wk, 4wk | Control group | No intervention | ②；⑧；⑨；⑫ |

| Study | | | Participants | | | | Intervention | | | | | Control group | | Outcomes |
| --- | --- | --- | --- | --- | --- | --- | --- | --- | --- | --- | --- | --- | --- | --- |
| Song,2018 | China | RCT | MCI | Hospital | IG:56.73±7.61 CG:57.50±6.47 | IG:30(14/16) CG:30(17/13) | VR combining with traditional cognitive training | VR Comprehensive Cognitive Game | semi-immersion | Qingdao Hailan Rehabilitation Equipment Digital Evaluation and Interactive Training System, Display | 15min, 5times/wk, 4wk | Active control group | Regular cognitive training | ①；② |
| Sun et al.,2021a | China | RCT | Dementia | Hospital | IG:78.58±6.26 CG:79.01±3.94 | IG:28(9/19) CG:28(11/17) | VR scenario interactive games | VR comprehensive cognitive games + Sport training | semi-immersion | VR scene interactive rehabilitation training system, display screen | 45min, 3times/wk, 24wk | Control group | Usual care | ①；⑩ |
| Sun et al.,2021b | China | RCT | MCI | Nursing institution | IG:77.33±4.00 CG:77.14±3.92 | IG:29(10/19) CG:28(11/17) | VR Ba Duan Jin training | VR Sport training | semi-immersion | Large screen plate | 50min, 3times/wk, 24wk | Control group | Usual care | ②；⑦；⑧；⑨；⑰；㉘ |
| Tarnanas et al.,2014 | Greece | RCT | MCI | Community | IG:70.5±4.3 CG:70.9±4.4 | IG:32(12/20) CG:33(13/21) | VR Museum cognitive training | Active control group | non-immersion | Computer screen, game handles | 90min, 2times/wk, 20wk | Control group | Regular daily activities | ①；③；④；⑨；㉙；㉚ |
| Thapa et al.,2020 | Korea | RCT | MCI | Health Care Center | IG:72.6±5.4 CG:72.7±5.6 | IG:34(6/28) CG:34(10/24) | VR Cognitive game training | VR Comprehensive Cognitive Game | Full immersion | Using Oculus VR head-mounted display, 2 wireless handles | 100min, 3times/wk, 8wk | Control group | Health education | ①；⑧；⑨；⑫；⑯；⑲；㉒ |
| Tominari et al.,2021 | Japan | RCT | MCI | Elder-care facilities and nursing homes | IG:85.1±7.25 CG:87.00±7.5 | IG:26(7/19) CG:26(5/21) | Recall therapy based on VR-360-degree panoramic images | VR Reminiscence Therapy | semi-immersion | 9.7-inch iPad | 30-45min, 1times/wk, 8wk | Active control group | Use printed panoramic photos | ① |

| Study | | | Participants | | | | Intervention | | | | | Control group | | Outcomes |
| --- | --- | --- | --- | --- | --- | --- | --- | --- | --- | --- | --- | --- | --- | --- |
| Ugur et al.,2020 | Turkey | RCT | Dementia | Nursing institution | IG:75±5.16 CG:71.5±3.54 | IG:16(11/5) CG:16(12/4) | Balanced and aerobic exercise training based on the Nintendo Wii device | VR Sport training | semi-immersion | Wireless remote control, balance board | 30min, 2times/wk, 6wk | Control group | Usual care | ①；⑫；⑮ |
| Wang et al.,2021 | China | RCT | MCI | Community | IG: 65.6±4.2 CG:63.9±4.0 | IG:21(11/10) CG:22(9/13) | Computerized Cognitive Remediation Therapy | Active control group | non-immersion | Computer | 30-40min, 5times/wk, 8wk | Control group | No intervention | ①；② |
| Xie et al.,2020 | China | RCT | Dementia | Medical-nursing combined institutions, elderly care institutions | IG:67.59±8.84 CG:68.79±9.57 | IG:28(13/15) CG:29(14/15) | Interactive games based on VR | VR Comprehensive Cognitive Game | Full immersion | HTC Vive pro head-mounted display, control handle | 30min, 2times/wk, 12wk | Active control group | Usual care, using a tablet computer for physical interaction game tasks | ① |
| Xue et al.,2021 | China | RCT | MCI | Hospital | NA | IG:30(24/6) CG:30(23/7) | Virtual reality technology-based rehabilitation training program | VR Comprehensive Cognitive Game | semi-immersion | Large screen plate | <30min,///,12wk | Active control group | Regular cognitive training | ② |
| Xu et al.,2020 | China | RCT | Dementia | Hospital | IG:66.80±2.89 CG:66.53±2.94 | IG:30(13/17) CG:30(14/16) | Comprehensive Cognitive Game | VR Comprehensive Cognitive Game | semi-immersion | Large screen plate | 30min, 5times/wk, 8wk | Control group | Usual care | ① |

| Study | | | Participants | | | | Intervention | | | | | Control group | | Outcomes |
| --- | --- | --- | --- | --- | --- | --- | --- | --- | --- | --- | --- | --- | --- | --- |
| Yang et al.,2019 | Taiwan | RCT | MCI | Retirement institutions and communities | IG:75.4±6.6 CG:81.7±7.2 | IG:33(8/25) CG:33(6/27) | Virtual Interactive Working Memory Training | VR Comprehensive Cognitive Game | non-immersion | Tablet pc | 45min, 3times/wk, 12wk | Active control group | Using a tablet for regular cognitive training | ①；②；③ |
| Yang et al.,2022 | Korea | RCT | MCI | Community | IG:72.5±5 CG:72.6±5.6 | IG:33(13/20) CG:33(6/27) | Virtual Reality Cognitive Training (VRCT) | VR Comprehensive Cognitive Game | Full immersion | Using Oculus VR head-mounted display, 2 wireless handles | 100min, 3times/wk, 8wk | Control group | Health education | ①；⑧；⑲ |
| Yang et al.,2022 | Korea | RCT | MCI | Community | IG:67.9±3.6 CG:72.6±5.6 | IG:33(3/30) CG:33(6/27) | Exercise training | Active control group | non-immersion | Conventional fitness equipment、Heart rate monitoring equipment | 60min, 2times/wk, 12wk | Control group | Health education | ①；⑧；⑲ |
| Zheng et al.,2021 | China | RCT | Dementia | Senior day care centers and nursing homes | IG:81.74±5.79 CG:84.26±5.48 | IG:18(3/15) CG:20(6/14) | Xbox 360 Kinect Game Training | VR Comprehensive Cognitive Game | semi-immersion | Kinect Sensor, Console | 60min, 5times/wk, 8wk | Control group | Usual care | ① |
| Zheng et al.,2024 | China | RCT | MCI | Nursing institution | IG:80.88±8.75 CG:79.52±9.61 | IG:33(12/21) CG:33(18/15) | Rehabilitation training for daily life activities based on virtual reality | VR Supermarket Training | Full immersion | Head-mounted VR display, handheld controller and tracker | 45min, 2times/wk, 12wk | Control group | Usual care | ① |

*Note:* NA: not available；IG: intervention group；CG: control group; VR: virtual reality；VR-CCG: VR Comprehensive Cognitive Game; VR-SPT: VR Sport Training; VR-MT: VR Reminiscence Therapy; ①MMSE: Mini-Mental State Examination；②MoCA: Montreal Cognitive Assessment；③DST-Backward: Digit Span Test - Backward；④DST-Forward: Digit Span Test - Forward；⑤FAB: Frontal Assessment Battery；⑥VFT: Verbal Fluency Test；⑦DSST: Digit Symbol Substitution Test；⑧TMT-A: Trail Making Test Part A；⑨TMT-B: Trail Making Test Part B；⑩TUG: Timed Up and Go Test；⑪iTUG: Instrumented Timed Up and Go Test；⑫GS: Gait Speed Test；⑬6mwt: 6-Minute Walk Test；⑭BBS: Berg Balance Scale；⑮5STS: Five Times Sit-to-Stand Test；⑯8-Foot Up and Go: 8-Foot Up and Go Test (=2.44m Walk)；⑰SPPB: Short Physical Performance Battery (Containing TST sub-items)；⑱TST: Tandem Stand Test (Belonging to SPPB)；⑲SDST: Symbol Digit Substitution Test；⑳CDT: Clock Drawing Test；㉑FSST: Four Square Step Test；㉒HGS-L/R: Hand Grip Strength - Left/Right；㉓Tinetti : Tinetti Performance Oriented Mobility Assessment；㉔Tinetti Balance: Tinetti Performance Oriented Mobility Assessment - Balance Subscale；㉕Tinetti Gait: Tinetti Performance Oriented Mobility Assessment - Gait Subscale；㉖VLT-Immediate: Verbal Learning Test - Immediate Recall；㉗VLT-Delayed: Verbal Learning Test - Delayed Recall；㉘RBMT-II: Rivermead Behavioural Memory Test - Second Edition；㉙SCWT: Stroop Color-Word Test；㉚RAVLT：Rey Auditory Verbal Learning Test。

# Appendix 5 The list for the included studies

1.	Tartaglia MC, Rosen HJ, Miller BL. Neuroimaging in dementia. Neurotherapeutics. 2011;8(1):82-92.

2.	Kelley BJ, Petersen RC. Alzheimer's disease and mild cognitive impairment. Neurol Clin. 2007;25(3):577-609, v.

3.	Organization WH. Dementia 2025 [Available from: https://www.who.int/news-room/fact-sheets/detail/dementia.

4.	Estimation of the global prevalence of dementia in 2019 and forecasted prevalence in 2050: an analysis for the Global Burden of Disease Study 2019. Lancet Public Health. 2022;7(2):e105-e25.

5.	Salari N, Lotfi F, Abdolmaleki A, Heidarian P, Rasoulpoor S, Fazeli J, et al. The global prevalence of mild cognitive impairment in geriatric population with emphasis on influential factors: a systematic review and meta-analysis. BMC Geriatr. 2025;25(1):313.

6.	Nichols E, Steinmetz JD, Vollset SE, Fukutaki K, Chalek J, Abd-Allah F, et al. Estimation of the global prevalence of dementia in 2019 and forecasted prevalence in 2050: an analysis for the Global Burden of Disease Study 2019. The Lancet Public Health. 2022;7(2):e105-e25.

7.	Livingston G, Huntley J, Sommerlad A, Ames D, Ballard C, Banerjee S, et al. Dementia prevention, intervention, and care: 2020 report of the Lancet Commission. The Lancet. 2020;396(10248):413-46.

8.	Amrapala A, Sabé M, Solmi M, Maes M. Neuropsychiatric disturbances in mild cognitive impairment: A scientometric analysis. Ageing Res Rev. 2023;92:102129.

9.	Arevalo-Rodriguez I, Smailagic N, Roqué-Figuls M, Ciapponi A, Sanchez-Perez E, Giannakou A, et al. Mini-Mental State Examination (MMSE) for the early detection of dementia in people with mild cognitive impairment (MCI). Cochrane Database Syst Rev. 2021;7(7):Cd010783.

10.	Petersen RC. Mild Cognitive Impairment. Continuum (Minneap Minn). 2016;22(2 Dementia):404-18.

11.	Hugo J, Ganguli M. Dementia and cognitive impairment: epidemiology, diagnosis, and treatment. Clin Geriatr Med. 2014;30(3):421-42.

12.	Orgeta V, McDonald KR, Poliakoff E, Hindle JV, Clare L, Leroi I. Cognitive training interventions for dementia and mild cognitive impairment in Parkinson's disease. Cochrane Database Syst Rev. 2020;2(2):Cd011961.

13.	Dale W, Hougham GW, Hill EK, Sachs GA. High interest in screening and treatment for mild cognitive impairment in older adults: A pilot study. J Am Geriatr Soc. 2006;54(9):1388-94.

14.	Odawara T. Cautious notification and continual monitoring of patients with mild cognitive impairment. Psychogeriatrics. 2012;12(2):131-2.

15.	Eshkoor SA, Hamid TA, Mun CY, Ng CK. Mild cognitive impairment and its management in older people. Clin Interv Aging. 2015;10:687-93.

16.	Lasaponara S, Marson F, Doricchi F, Cavallo M. A Scoping Review of Cognitive Training in Neurodegenerative Diseases via Computerized and Virtual Reality Tools: What We Know So Far. Brain Sci. 2021;11(5).

17.	Salim NA, Taylor J. Efficacy of Virtual Reality in Managing Pain for Patients in Palliative Care: A Systematic Review and Meta-Analysis. J Pain Symptom Manage. 2026;71(1):e1-e17.

18.	Papaioannou T, Voinescu A, Petrini K, Stanton Fraser D. Efficacy and Moderators of Virtual Reality for Cognitive Training in People with Dementia and Mild Cognitive Impairment: A Systematic Review and Meta-Analysis. J Alzheimers Dis. 2022;88(4):1341-70.

19.	Fordell H, Wåhlin A, Ekman U, Malm J, Lenfeldt N. Rehabilitation in Chronic Neglect Strengthens Functional Connectivity Between Nodes of the Dorsal Attention Network. Archives of Physical Medicine and Rehabilitation. 2018;99(10):e10.

20.	Gómez-Cáceres B, Cano-López I, Aliño M, Puig-Perez S. Effectiveness of virtual reality-based neuropsychological interventions in improving cognitive functioning in patients with mild cognitive impairment: A systematic review and meta-analysis. Clin Neuropsychol. 2023;37(7):1337-70.

21.	Goumopoulos C, Skikos G, Frounta M. Feasibility and Effects of Cognitive Training with the COGNIPLAT Game Platform in Elderly with Mild Cognitive Impairment: pilot Randomized Controlled Trial. Games for health journal. 2023;12(5):414‐25.

22.	Ip WK, Soar J, James C, Wang SY, Fong KNK. Virtual reality game-based training for preventing falls among community-dwelling older adults with mild cognitive impairment: a pilot randomized control trial study. Virtual Reality. 2025;29(1).

23.	Padala KP, Padala PR, Malloy TR, Geske JA, Dubbert PM, Dennis RA, et al. Wii-fit for improving gait and balance in an assisted living facility: a pilot study. Journal of aging research. 2012;2012.

24.	Amjad I, Toor H, Niazi IK, Pervaiz S, Jochumsen M, Shafique M, et al. Xbox 360 Kinect Cognitive Games Improve Slowness, Complexity of EEG, and Cognitive Functions in Subjects with Mild Cognitive Impairment: A Randomized Control Trial. Games for Health Journal. 2019;8(2):144-52.

25.	Arshad H, Khattak HG, Anwar K. Effect of exergames by using Xbox 360 Kinect on cognition of older adults with mild cognitive impairment. Rawal Medical Journal. 2023;48(4):994-8.

26.	Kang JM, Kim N, Lee SY, Woo SK, Park G, Yeon BK, et al. Effect of Cognitive Training in Fully Immersive Virtual Reality on Visuospatial Function and Frontal-Occipital Functional Connectivity in Predementia: Randomized Controlled Trial. J Med Internet Res. 2021;23(5):e24526.

27.	Hagovska M, Olekszyova Z. Impact of the combination of cognitive and balance training on gait, fear and risk of falling and quality of life in seniors with mild cognitive impairment. Geriatr Gerontol Int. 2016;16(9):1043-50.

28.	Schwenk M, Sabbagh M, Lin I, Morgan P, Grewal GS, Mohler J, et al. Sensor-based balance training with motion feedback in people with mild cognitive impairment. J Rehabil Res Dev. 2016;53(6):945-58.

29.	Hsieh CC, Lin PS, Hsu WC, Wang JS, Huang YC, Lim AY, et al. The Effectiveness of a Virtual Reality-Based Tai Chi Exercise on Cognitive and Physical Function in Older Adults with Cognitive Impairment. Dement Geriatr Cogn Disord. 2018;46(5-6):358-70.

30.	Moulaei K, Sharifi H, Bahaadinbeigy K, Dinari F. Efficacy of virtual reality-based training programs and games on the improvement of cognitive disorders in patients: a systematic review and meta-analysis. BMC Psychiatry. 2024;24(1):116.

31.	Yu J, Song J, Shen Q. Effects of fully immersive virtual reality training on cognitive function in patients with mild cognitive impairment: a systematic review and meta-analysis. Front Hum Neurosci. 2024;18:1467697.

32.	Yang Q, Zhang L, Chang F, Yang H, Chen B, Liu Z. Virtual Reality Interventions for Older Adults With Mild Cognitive Impairment: Systematic Review and Meta-Analysis of Randomized Controlled Trials. J Med Internet Res. 2025;27:e59195.

33.	Ren Y, Wang Q, Liu H, Wang G, Lu A. Effects of immersive and non-immersive virtual reality-based rehabilitation training on cognition, motor function, and daily functioning in patients with mild cognitive impairment or dementia: A systematic review and meta-analysis. Clin Rehabil. 2024;38(3):305-21.

34.	Zhao YN, Feng H, Wu XY, Du Y, Yang XF, Hu MY, et al. Effectiveness of Exergaming in Improving Cognitive and Physical Function in People With Mild Cognitive Impairment or Dementia: Systematic Review. Jmir Serious Games. 2020;8(2).

35.	Buele J, Avilés-Castillo F, Del-Valle-Soto C, Varela-Aldás J, Palacios-Navarro G. Effects of a dual intervention (motor and virtual reality-based cognitive) on cognition in patients with mild cognitive impairment: a single-blind, randomized controlled trial (vol 21, 130, 2024). Journal of Neuroengineering and Rehabilitation. 2024;21(1).

36.	Choi W, Lee S. The Effects of Virtual Kayak Paddling Exercise on Postural Balance, Muscle Performance, and Cognitive Function in Older Adults with Mild Cognitive Impairment: A Randomized Controlled Trial. J Aging Phys Act. 2019;27(4):861-70.

37.	Delbroek T, Vermeylen W, Spildooren J. The effect of cognitive-motor dual task training with the biorescue force platform on cognition, balance and dual task performance in institutionalized older adults: a randomized controlled trial. J Phys Ther Sci. 2017;29(7):1137-43.

38.	Tominari M, Uozumi R, Becker C, Kinoshita A, Castelnuovo G. Reminiscence therapy using virtual reality technology affects cognitive function and subjective well-being in older adults with dementia. Cogent Psychology. 2021;8(1).

39.	Salanti G, Higgins JP, Ades AE, Ioannidis JP. Evaluation of networks of randomized trials. Stat Methods Med Res. 2008;17(3):279-301.

40.	Ades AE, Sculpher M, Sutton A, Abrams K, Cooper N, Welton N, et al. Bayesian methods for evidence synthesis in cost-effectiveness analysis. Pharmacoeconomics. 2006;24(1):1-19.

41.	Hutton B, Salanti G, Caldwell DM, Chaimani A, Schmid CH, Cameron C, et al. The PRISMA extension statement for reporting of systematic reviews incorporating network meta-analyses of health care interventions: checklist and explanations. Ann Intern Med. 2015;162(11):777-84.

42.	Sterne JAC, Savović J, Page MJ, Elbers RG, Blencowe NS, Boutron I, et al. RoB 2: a revised tool for assessing risk of bias in randomised trials. Bmj. 2019;366:l4898.

43.	Nikolakopoulou A, Higgins JPT, Papakonstantinou T, Chaimani A, Del Giovane C, Egger M, et al. CINeMA: An approach for assessing confidence in the results of a network meta-analysis. PLoS Med. 2020;17(4):e1003082.

44.	Borenstein M, Hedges LV, Higgins JP, Rothstein HR. A basic introduction to fixed-effect and random-effects models for meta-analysis. Res Synth Methods. 2010;1(2):97-111.

45.	Langan D, Higgins JPT, Jackson D, Bowden J, Veroniki AA, Kontopantelis E, et al. A comparison of heterogeneity variance estimators in simulated random-effects meta-analyses. Res Synth Methods. 2019;10(1):83-98.

46.	Noma H, Hamura Y, Sugasawa S, Furukawa TA. Improved methods to construct prediction intervals for network meta-analysis. Res Synth Methods. 2023;14(6):794-806.

47.	Yang JG, Thapa N, Park HJ, Bae S, Park KW, Park JH, et al. Virtual Reality and Exercise Training Enhance Brain, Cognitive, and Physical Health in Older Adults with Mild Cognitive Impairment. Int J Environ Res Public Health. 2022;19(20).

48.	Shim S, Yoon BH, Shin IS, Bae JM. Network meta-analysis: application and practice using Stata. Epidemiol Health. 2017;39:e2017047.

49.	Borenstein M. How to understand and report heterogeneity in a meta-analysis: The difference between I-squared and prediction intervals. Integrative Medicine Research. 2023;12(4):101014.

50.	Bhatnagar N, Lakshmi PV, Jeyashree K. Multiple treatment and indirect treatment comparisons: An overview of network meta-analysis. Perspect Clin Res. 2014;5(4):154-8.

51.	Puerto Nino AK, Brignardello-Petersen R. How To Read a Network Meta-analysis. Eur Urol Focus. 2023;9(5):701-4.

52.	Lu LJ, Xiong K, Yuan SL, Che BW, Zhai JC, Wu CC, et al. Surgical approaches to varicocele: a systematic review and network meta-analysis. Asian J Androl. 2025;27(6):728-37.

53.	Tan CJ, Shufelt T, Behan E, Chantara J, Koomsri C, Gordon AJ, et al. Comparative effectiveness of psychosocial interventions in adults with harmful use of alcohol: a systematic review and network meta-analysis. Addiction. 2023;118(8):1414-29.

54.	Borenstein M, Higgins JPT. Meta-Analysis and Subgroups. Prevention Science. 2013;14(2):134-43.

55.	Ding L. The Effect of Virtual Supermarket Cognitive Assessment and Training System on the Elderly with Mild Cognitive Impairment [Master]: Jilin University; 2023.

56.	Lim EH, Kim DS, Won YH, Park SH, Seo JH, Ko MH, et al. Effects of Home Based Serious Game Training (Brain Talk) in the Elderly With Mild Cognitive Impairment: Randomized, a Single-Blind, Controlled Trial. Brain Neurorehabil. 2023;16(1):e4.

57.	Man DW, Chung JC, Lee GY. Evaluation of a virtual reality-based memory training programme for Hong Kong Chinese older adults with questionable dementia: a pilot study. Int J Geriatr Psychiatry. 2012;27(5):513-20.

58.	Manenti R, Gobbi E, Baglio F, Macis A, Ferrari C, Pagnoni I, et al. Effectiveness of an Innovative Cognitive Treatment and Telerehabilitation on Subjects With Mild Cognitive Impairment: A Multicenter, Randomized, Active-Controlled Study. Front Aging Neurosci. 2020;12:585988.

59.	Oliveira J, Gamito P, Souto T, Conde R, Ferreira M, Corotnean T, et al. Virtual Reality-Based Cognitive Stimulation on People with Mild to Moderate Dementia due to Alzheimer's Disease: A Pilot Randomized Controlled Trial. Int J Environ Res Public Health. 2021;18(10).

60.	Park JH, Liao Y, Kim DR, Song S, Lim JH, Park H, et al. Feasibility and Tolerability of a Culture-Based Virtual Reality (VR) Training Program in Patients with Mild Cognitive Impairment: A Randomized Controlled Pilot Study. Int J Environ Res Public Health. 2020;17(9).

61.	Park JS, Jung YJ, Lee G. Virtual Reality-Based Cognitive-Motor Rehabilitation in Older Adults with Mild Cognitive Impairment: A Randomized Controlled Study on Motivation and Cognitive Function. Healthcare. 2020;8(3).

62.	Song J. Virtual reality technology in the application of vascular cognitive impairment with no dementia and remote rehabilitation [Master]: Qingdao University; 2018.

63.	Thapa N, Park HJ, Yang JG, Son H, Jang M, Lee J, et al. The effect of a virtual reality-based intervention program on cognition in older adults with mild cognitive impairment: A randomized control trial. Journal of Clinical Medicine. 2020;9(5).

64.	Xie X, Nong Q, Nong D, Pan X, Liu S, Li X, et al. Application of immersive virtual reality technique to cognitive training in senile dementia patients. Guangxi Medical Journal. 2020;42(20):2717-20.

65.	Xue J, Qin J. Analysis of the Application Effect of Virtual Reality Technology in the Rehabilitation Training of Patients with Mild Cognitive Impairment. Chinese General Practice. 2021;24(S1):110-2.

66.	Xu Q, Liu X, Li P, Deng Z, Ji P. Efficacy of cognitive treadmill training on mild to moderate Alzheimer 's disease. Chinese Journal of Gerontology. 2020;40(13):2813-4.

67.	Yang HL, Chu H, Kao CC, Chiu HL, Tseng IJ, Tseng P, et al. Development and effectiveness of virtual interactive working memory training for older people with mild cognitive impairment: a single-blind randomised controlled trial. Age Ageing. 2019;48(4):519-25.

68.	Hu D, He J, Ding Y, Xu J, Zhu H. Application Effect of Virtual Reality Technology in the Pulmonary Rehabilitation Program for Elderly COPD Patients Complicated with Mild Cognitive Impairment. Practical Journal of Cardiac Cerebral Pneumal and Vascular Disease. 2018;26(06):85-8.

69.	Li AY, Li JW, Wu W, Zhao JJ, Qiang Y. Effect of Virtual Reality Training on Cognitive Function and Motor Performance in Older Adults With Cognitive Impairment Receiving Health Care: A Randomized Controlled Trial. International Journal of Human-Computer Interaction. 2024;40(22):7755-72.

70.	Liao YY, Hsuan Chen I, Lin YJ, Chen Y, Hsu WC. Effects of virtual reality-based physical and cognitive training on executive function and dual-task gait performance in older adults with mild cognitive impairment: A randomized control trial. Frontiers in Aging Neuroscience. 2019;10(JUL).

71.	Li C, Shang Y, Wang L, Yang Y, Shen Y. Research on the influence of computer cognitive training on the cognitive function of the elderly with mild cognitive impairment. Chinese Journal of Nursing. 2021;56(05):667-73.

72.	Sun Z, Wang T, Ma J, Zhang N, Pan L, Dong Y. Application of virtual reality situational interactive training in the rehabilitation treatment of patients with Alzheimer′s disease in nursing home. Journal of Hebei Medical University. 2021;42(02):222-7.

73.	Liu CL, Cheng FY, Wei MJ, Liao YY. Effects of Exergaming-Based Tai Chi on Cognitive Function and Dual-Task Gait Performance in Older Adults With Mild Cognitive Impairment: A Randomized Control Trial. Frontiers in Aging Neuroscience. 2022;14.

74.	Sun Z, Ma J, Gu X, Ouyang G, Zhang N, Chen X, et al. Baduanjin training based on virtual reality can relieve mild cognitive impairment in the elderly. Chinese Journal of Physical Medicine and Rehabilitation. 2021;43(04):322-6.

75.	Ugur F, Sertel M. The Effect of Virtual Reality Applications on Balance and Gait Speed in Individuals with Alzheimer Dementia: a Pilot Study. Topics in geriatric rehabilitation. 2020;36(4):221‐9.

76.	Liao YY, Tseng HY, Lin YJ, Wang CJ, Hsu WC. Using virtual reality-based training to improve cognitive function, instrumental activities of daily living and neural efficiency in older adults with mild cognitive impairment. Eur J Phys Rehabil Med. 2020;56(1):47-57.

77.	Li S, Li W, Zhao Y, Tan Y, Bai S. Effects of dual-task training based on serious games. Journal of Nursing Science. 2023;38(24):88-91.

78.	Hughes TF, Flatt JD, Fu B, Butters MA, Chang CC, Ganguli M. Interactive video gaming compared with health education in older adults with mild cognitive impairment: a feasibility study. Int J Geriatr Psychiatry. 2014;29(9):890-8.

79.	Chan JYC, Liu J, Chan ATC, Tsoi KKF. Exergaming and cognitive functions in people with mild cognitive impairment and dementia: a meta-analysis. NPJ Digit Med. 2024;7(1):154.

80.	Baldimtsi E, Mouzakidis C, Karathanasi EM, Verykouki E, Hassandra M, Galanis E, et al. Effects of Virtual Reality Physical and Cognitive Training Intervention On Cognitive Abilities of Elders with Mild Cognitive Impairment. J Alzheimers Dis Rep. 2023;7(1):1475-90.

81.	Tsai CL, Pai MC, Ukropec J, Ukropcová B. Distinctive Effects of Aerobic and Resistance Exercise Modes on Neurocognitive and Biochemical Changes in Individuals with Mild Cognitive Impairment. Curr Alzheimer Res. 2019;16(4):316-32.

82.	Broadhouse KM, Singh MF, Suo C, Gates N, Wen W, Brodaty H, et al. Hippocampal plasticity underpins long-term cognitive gains from resistance exercise in MCI. Neuroimage Clin. 2020;25:102182.

83.	Barha CK, Galea LA, Nagamatsu LS, Erickson KI, Liu-Ambrose T. Personalising exercise recommendations for brain health: considerations and future directions. Br J Sports Med. 2017;51(8):636-9.

84.	Duclos M, Tabarin A. Exercise and the Hypothalamo-Pituitary-Adrenal Axis. Front Horm Res. 2016;47:12-26.

85.	Feter N, Schaun GZ, Smith EC, Cassuriaga J, Alt R, Redig L, et al. High-velocity resistance training improves executive function in mobility-limited older adults. Arch Gerontol Geriatr. 2023;114:105081.

86.	Kong J, Xie Y, Fan R, Wang Q, Luo Y, Dong P. Exercise orchestrates systemic metabolic and neuroimmune homeostasis via the brain-muscle-liver axis to slow down aging and neurodegeneration: a narrative review. Eur J Med Res. 2025;30(1):475.

87.	Cooper RA, Ding D, Simpson R, Fitzgerald SG, Spaeth DM, Guo S, et al. Virtual reality and computer-enhanced training applied to wheeled mobility: an overview of work in Pittsburgh. Assist Technol. 2005;17(2):159-70.

88.	Gourlay D, Lun KC, Lee YN, Tay J. Virtual reality for relearning daily living skills. Int J Med Inform. 2000;60(3):255-61.

89.	Colcombe S, Kramer AF. Fitness effects on the cognitive function of older adults: a meta-analytic study. Psychol Sci. 2003;14(2):125-30.

90.	Northey JM, Cherbuin N, Pumpa KL, Smee DJ, Rattray B. Exercise interventions for cognitive function in adults older than 50: a systematic review with meta-analysis. Br J Sports Med. 2018;52(3):154-60.

91.	Wilke J, Giesche F, Klier K, Vogt L, Herrmann E, Banzer W. Acute Effects of Resistance Exercise on Cognitive Function in Healthy Adults: A Systematic Review with Multilevel Meta-Analysis. Sports Med. 2019;49(6):905-16.

92.	Anderson-Hanley C, Arciero PJ, Brickman AM, Nimon JP, Okuma N, Westen SC, et al. Exergaming and older adult cognition: a cluster randomized clinical trial. Am J Prev Med. 2012;42(2):109-19.

93.	Bastioli G, Arnold JC, Mancini M, Mar AC, Gamallo-Lana B, Saadipour K, et al. Voluntary Exercise Boosts Striatal Dopamine Release: Evidence for the Necessary and Sufficient Role of BDNF. J Neurosci. 2022;42(23):4725-36.

94.	Alwashmi K, Meyer G, Rowe F, Ward R. Enhancing learning outcomes through multisensory integration: A fMRI study of audio-visual training in virtual reality. Neuroimage. 2024;285:120483.

95.	Kim H, Lee IK. Studying the Effects of Congruence of Auditory and Visual Stimuli on Virtual Reality Experiences. IEEE Trans Vis Comput Graph. 2022;28(5):2080-90.

96.	Wu J, Nie S, Li C, Wang X, Peng Y, Shang J, et al. Sound-localization-related activation and functional connectivity of dorsal auditory pathway in relation to demographic, cognitive, and behavioral characteristics in age-related hearing loss. Front Neurosci. 2024;18:1353413.

97.	Riva G, Serino S, Di Lernia D, Pagnini F. Regenerative Virtual Therapy: The Use of Multisensory Technologies and Mindful Attention for Updating the Altered Representations of the Bodily Self. Front Syst Neurosci. 2021;15:749268.

98.	Szczepocka E, Mokros Ł, Kazmierski J, Nowakowska K, Łucka A, Antoszczyk A, et al. The Effectiveness of Virtual Reality-Based Training on Cognitive, Social, and Physical Functioning in High-Functioning Older Adults (CoSoPhy FX): 2-Arm, Parallel-Group Randomized Controlled Trial. JMIR Res Protoc. 2024;13:e53261.

99.	Kalantari S, Xu TB, Mostafavi A, Kim B, Dilanchian A, Lee A, et al. Using Immersive Virtual Reality to Enhance Social Interaction Among Older Adults: A Cross-Site Investigation. Innov Aging. 2023;7(4):igad031.

100.	Wei X, Huang C, Ding X, Zhou Z, Zhang Y, Feng X, et al. Effect of virtual reality training on dual-task performance in older adults: a systematic review and meta-analysis. J Neuroeng Rehabil. 2025;22(1):141.

# Appendix 6 Risk of bias

Figure S1 The overall risk of bias for all included studies

Figure S2 The risk of bias for each study

# Appendix 7 The analysis for outcomes

## 7.1 Traditional pairwise meta-analysis

### 7.1.1 Global cognition

Figure S3 VR-CCG vs CG

Figure S4 VR-CCG+SPT vs CG

Figure S5 VR-SPT vs CG

Figure S6 VR-CCG vs ACG

Figure S7 VR-CCG+SPT vs ACG

Figure S8 VR-SPT vs ACG

### 7.1.2 Primary analysis for global cognition in MCI group

Figure S9 VR-CCG vs CG

Figure S10 VR-CCG+SPT vs CG

Figure S11 VR-CCG vs ACG

Figure S12 VR-CCG+SPT vs ACG

### 7.1.3 Primary analysis for global cognition in AD group

Figure S13 VR-CCG vs CG

### 7.1.4 Executive function

Figure S14 VR-CCG vs CG

Figure S15 VR-CCG+SPT vs CG

Figure S16 VR-CCG vs ACG

Figure S17 VR-CCG+SPT vs ACG

### 7.1.5 Primary analysis for executive function in MCI group

Figure S18 VR-CCG vs CG

Figure S19 VR-CCG+SPT vs CG

Figure S20 VR-CCG vs ACG

Figure S21 VR-CCG+SPT vs ACG

### 7.1.6 Motor function

Figure S22 VR-CCG+SPT vs CG

Figure S23 VR-SPT vs CG

Figure S24 VR-CCG+SPT vs ACG

### 7.1.7 Primary analysis for motor function in MCI group

Figure S25 VR-CCG+SPT vs CG

Figure S26 VR-SPT vs CG

Figure S27 VR-CCG+SPT vs ACG

*Note:* the horizontal black line segment at the bottom of the forest plot represents the 95% prediction interval.

## 7. 2 Network analysis

### 7.2.1 Global cognition

（1）Global inconsistency test

**χ^2^ = 4.06**

***p* = 0.2554**

No evidence for the existence of significant global inconsistency

（2）Local inconsistency test

Figure S28 Local inconsistency test

（3）The ranking of effects for global cognition

| Treatment | SUCRA | Probability of Best (%) | Mean Rank |
| --- | --- | --- | --- |
| VR-SPT | 90 | 66.6 | 1.6 |
| VR-CCG+SPT | 64.4 | 9.0 | 2.8 |
| VR-CCG | 63.2 | 5.2 | 2.8 |
| VR-MT | 45.7 | 19.3 | 3.7 |
| ACG | 25 | 0.0 | 4.7 |
| CG | 11.8 | 0.0 | 5.4 |

Table S13 The ranking of effects of different VR interventions on global cognition

Figure S29 Ranking plot for global cognition

（4）The comparison-adjusted funnel plot for global cognition

Figure S30 The comparison-adjusted funnel plot for global cognition

### 7.2.2 Primary analysis for global cognition in MCI group

（1）Global inconsistency test

**χ^2^ = 10.59**

***p* = 0.0142**

No evidence for the existence of significant global inconsistency

（2）Local inconsistency test

Figure S31 Local inconsistency test

（3）Network graph

Figure S32 Network graph for global cognition in MCI

（4）The ranking of effects for global cognition in MCI

| Treatment | SUCRA | Probability of Best (%) | Mean Rank |
| --- | --- | --- | --- |
| VR-SPT | 99.6 | 97.8 | 1.0 |
| VR-CCG | 61.3 | 0.2 | 2.9 |
| VR-CCG+SPT | 61.3 | 0.2 | 3.0 |
| VR-MT | 41.1 | 1.9 | 3.9 |
| ACG | 23.2 | 0.0 | 4.8 |
| CG | 13.5 | 0.0 | 5.3 |

Table S14 The ranking of effects of different VR interventions on global cognition in MCI

Figure S33 Ranking plot for global cognition in MCI

（5）Relative effect sizes combined with SUCRA values of different interventions according to network meta-analysis for global cognitive function in MCI

Figure S34 contingency tables for global cognition in MCI

*No*te: the 95% confidence interval (indicated by parentheses) and the 95% prediction interval [indicated by square brackets] are presented.

（6）The comparison-adjusted funnel plot for global cognition in MCI

Figure S35 The comparison-adjusted funnel plot for global cognition in MCI

### 7.2.3 Primary analysis for global cognition in AD group

（1）Global inconsistency test

**χ^2^ = 2.73**

***p* = 0.0986**

No evidence for the existence of significant global inconsistency

（2）Local inconsistency test

Figure S36 Local inconsistency test

（3）Network graph

Figure S37 Network graph for global cognition in AD

（4）The ranking of effects for global cognition in AD

| Treatment | SUCRA | Probability of Best (%) | Mean Rank |
| --- | --- | --- | --- |
| VR-CCG+SPT | 84.3 | 57.7 | 1.6 |
| VR-CCG | 81.2 | 33.8 | 1.8 |
| ACG | 50.5 | 7.9 | 3.0 |
| CG | 17.4 | 0.0 | 4.3 |
| VR-SPT | 16.6 | 0.7 | 4.3 |

Table S15 The ranking of effects of different VR interventions on global cognition in AD

Figure S38 Ranking plot for global cognition in AD

（5）Relative effect sizes combined with SUCRA values of different interventions according to network meta-analysis for global cognitive function in AD

Figure S39 contingency tables for global cognition in AD

*No*te: the 95% confidence interval (indicated by parentheses) and the 95% prediction interval [indicated by square brackets] are presented.

（6）The comparison-adjusted funnel plot for global cognition in AD

Figure S40 The comparison-adjusted funnel plot for global cognition in AD

### 7.2.4 Executive function

（1）Global inconsistency test

χ^2^ =4.88

*p* =0.1804

No evidence for the existence of significant global inconsistency

（2）Local inconsistency test

Figure S41 Local inconsistency test

（3）The ranking of effects for executive function

| Treatment | SUCRA | Probability of Best (%) | Mean Rank |
| --- | --- | --- | --- |
| VR-SPT | 98.1 | 94.8 | 1.1 |
| VR-CCG | 68.8 | 3.8 | 2.3 |
| VR-CCG+SPT | 50.8 | 1.3 | 2.9 |
| CG | 17.2 | 0.0 | 4.4 |
| ACG | 15.2 | 0.0 | 4.4 |

Table S16 The ranking of effects of different VR interventions on executive function

Figure S42 Ranking plot for executive function

（5）The comparison-adjusted funnel plot for executive function

Figure S43 The comparison-adjusted funnel plot for executive function

### 7.2.5 Primary analysis for executive function in MCI group

（1）Global inconsistency test

**χ^2^ = 4.35**

***p* = 0.226**

No evidence for the existence of significant global inconsistency

（2）Local inconsistency test

Figure S44 Local inconsistency test

（3）Network graph

Figure S45 Network graph for executive function in MCI

（4）The ranking of effects for executive function in MCI

| Treatment | SUCRA | Probability of Best (%) | Mean Rank |
| --- | --- | --- | --- |
| VR-SPT | 97.7 | 93.6 | 1.1 |
| VR-CCG | 69.6 | 4.9 | 2.2 |
| VR-CCG+SPT | 49.9 | 1.4 | 3.0 |
| CG | 16.5 | 0.0 | 4.3 |
| ACG | 16.4 | 0.0 | 4.4 |

Table S17 The ranking of effects of different VR interventions on executive function in MCI

Figure S46 Ranking plot for executive function in MCI

（5）Relative effect sizes combined with SUCRA values of different interventions according to network meta-analysis for executive function in MCI

Figure S47 contingency tables for executive function in MCI

*No*te: the 95% confidence interval (indicated by parentheses) and the 95% prediction interval [indicated by square brackets] are presented.

（6）The comparison-adjusted funnel plot for executive function in MCI

Figure S48 The comparison-adjusted funnel plot for executive function in MCI

### 7.2.6 Motor function

（1）Global inconsistency test

χ^2^ =0.68

*p* =0.4112

No evidence for the existence of significant global inconsistency

（2）Local inconsistency test

Figure S49 Local inconsistency test

（3）The ranking of effects for motor function

| Treatment | SUCRA | Probability of Best (%) | Mean Rank |
| --- | --- | --- | --- |
| VR-CCG+SPT | 92.5 | 45.8 | 1.8 |
| VR-SPT | 56.3 | 24.0 | 2.4 |
| VR-CCG | 47.2 | 24.0 | 2.9 |
| ACG | 34.5 | 6.2 | 3.2 |
| CG | 19.5 | 0.0 | 4.7 |

Table S18 The ranking of effects of different VR interventions on motor function

Figure S50 Ranking plot for motor function

（4）The comparison-adjusted funnel plot for motor function

Figure S51 The comparison-adjusted funnel plot for motor function

### 7.2.7 Primary analysis for motor function in MCI group

（1）Global inconsistency test

No evidence for the existence of significant global inconsistency

（2）Local inconsistency test

Figure S52 Local inconsistency test

（3）Network graph

Figure S53 Network graph for motor function in MCI

（4）The ranking of effects for motor function in MCI

| Treatment | SUCRA | Probability of Best (%) | Mean Rank |
| --- | --- | --- | --- |
| VR-SPT | 79.3 | 44.9 | 1.9 |
| VR-CCG | 74.7 | 34.5 | 2.4 |
| VR-CCG+SPT | 39.1 | 17.9 | 2.6 |
| CG | 33.6 | 0.1 | 4.0 |
| ACG | 23.3 | 2.5 | 4.1 |

Table S19 The ranking of effects of different VR interventions on motor function in MCI

Figure S54 Ranking plot for motor function in MCI

（5）Relative effect sizes combined with SUCRA values of different interventions according to network meta-analysis for motor function in MCI

Figure S55 contingency tables for motor function in MCI

*No*te: the 95% confidence interval (indicated by parentheses) and the 95% prediction interval [indicated by square brackets] are presented.

（6）The comparison-adjusted funnel plot for motor function in MCI

Figure S56 The comparison-adjusted funnel plot for motor function in MCI

# Appendix 8 The results of meta regression analysis

Table S20 The classifications for VR types according to the frequency, duration per session, and the length of intervention.

| Number | Frequency | |
| --- | --- | --- |
| 1 | Low | 1-2 times/week |
| 2 | Moderate | 3-4 times/week |
| 3 | High | ≥5 times/week |
| Number | Duration per session | |
| 1 | Short | <30 minutes |
| 2 | Moderate-1 | 30-44 minutes |
| 3 | Moderate-2 | 45-59 minutes |
| 4 | long | ≥60 minutes |
| Number | Length of intervention | |
| 1 | Short | <8 weeks (2 months) |
| 2 | Moderate | 8-16 weeks (2 months-4 months) |
| 3 | long | ≥16 minutes (≥4 months) |

References:

1. Goumopoulos C, Skikos G, Frounta M. Feasibility and effects of cognitive training with the cogniplat game platform in elderly with mild cognitive impairment: Pilot randomized controlled trial. Journal article. *Games for health journal*. 2023;12(5):414‐425. [doi:10.1089/g4h.2023.0029][Medline:CN-02571838]
2. Arshad H, Khattak HG, Anwar K. Effect of exergames by using xbox 360 kinect on cognition of older adults with mild cognitive impairment. Article. *Rawal Medical Journal*. 2023;48(4):994-998. [doi:10.5455/rmj.20210821031024]
3. Buele J, Avilés-Castillo F, Del-Valle-Soto C, Varela-Aldás J, Palacios-Navarro G. Effects of a dual intervention (motor and virtual reality-based cognitive) on cognition in patients with mild cognitive impairment: A single-blind, randomized controlled trial (vol 21, 130, 2024). *Journal of Neuroengineering and Rehabilitation*. Aug 2024;21(1)149. [doi:10.1186/s12984-024-01443-5][Medline:WOS:001303582800001]
4. Li AY, Li JW, Wu W, Zhao JJ, Qiang Y. Effect of virtual reality training on cognitive function and motor performance in older adults with cognitive impairment receiving health care: A randomized controlled trial. *International Journal of Human-Computer Interaction*. Nov 2024;40(22):7755-7772.

The classification of virtual reality types based on the level of immersion

1、Immersive VR: This system fully immerses users by isolating their visual, auditory, and select tactile sensations from the physical environment and placing their perceptual awareness entirely within a computer-generated digital world. User tracking and interaction are achieved via head-mounted displays, handheld motion controllers, data gloves, or computer vision technology.

2、Semi-immersive VR: This technology projects virtual scenes onto one or more large screens, such as projection systems, panoramic displays, or high-resolution video walls, thereby embedding a computer-generated environment within the user's physical space. Users typically do not require fully enclosed headsets and may only need lightweight stereoscopic glasses to perceive three-dimensional imagery.

3、Non-immersive VR: Also known as desktop VR, this modality presents a 3D virtual environment on standard 2D displays like computer monitors, tablets, or smartphones. It provides a minimal level of immersion, as the user's visual and auditory attention remains shared with their physical surroundings. Interaction is facilitated through abstract commands via mice, keyboards, touchscreens, or game controllers (e.g., clicking, dragging), with no direct mapping between the user's body movements and actions within the virtual world.

Table S21 The results of meta regression

| Outcome Indicator | VR types | Covariates | Coef. | *p* | 95% CI |
| --- | --- | --- | --- | --- | --- |
| Cognitive function | VR-CCG | frequency | 0.31 | 0.30 | -0.29-0.91 |
|  |  | duration | -0.28 | 0.33 | -0.70-0.41 |
|  |  | length | 0.33 | 0.41 | -1.40-1.18 |
|  |  | Immersion level | -0.003 | 0.99 | -0.57-0.56 |
|  | VR-CCG+SPT | frequency | 0.42 | 0.19 | -0.26-1.11 |
|  |  | duration | -0.11 | 0.55 | -0.56-0.32 |
|  |  | length | 0.07 | 0.82 | -1.40-1.18 |
|  |  | Immersion level | -0.11 | 0.63 | -0.63-0.41 |
| Executive function | VR-CCG | frequency | 0.48 | 0.65 | -1.91-2.87 |
|  |  | duration | -0.55 | 0.51 | -2.39-1.28 |
|  |  | length | -0.65 | 0.75 | -5.34-4.04 |
|  |  | Immersion level | -0.49 | 0.65 | -2.83-1.84 |
|  | VR-CCG+SPT | frequency | 0.30 | 0.45 | -0.65-1.26 |
|  |  | duration | -0.20 | 0.45 | -0.83-0.42 |
|  |  | length | 0.42 | 0.46 | -0.94-1.81 |
|  |  | Immersion level | -0.12 | 0.70 | -0.83-0.59 |
| Motor function | VR-CCG+SPT | frequency | -0.96 | 0.19 | -2.79-0.86 |
|  |  | duration | -0.19 | 0.37 | -0.77-0.39 |
|  |  | length | 0.25 | 0.42 | -0.61-1.13 |
|  |  | Immersion level | 0.7 | 0.02 | 0.15-1.24 |

# Appendix 9 The distribution of key covariates in each VR intervention group

Table S22 The distribution of key covariates in each VR intervention group

| Outcome | Intervention | Participants, n | Mean Age, years (Mean ± SD) | male, % | Diagnosis | | VR Immersion | | | Intervention Dose | | |
| --- | --- | --- | --- | --- | --- | --- | --- | --- | --- | --- | --- | --- |
|  |  |  |  |  | MCI, % | Dementia, % | Full, n(%) | Semi, n(%) | Non, n(%) | Single duration (minutes) | Weekly frequency (times) | Total cycle (weeks) |
| Global Cognitive Function | VR-CCG（n=20） | 916 | 73.92 ± 5.12 | 40.6 | 80 | 20 | 6(30) | 7(35) | 7(35) | 42 | 3.26 | 8.25 |
|  | VR-CCG+SPT（n=11） | 495 | 76.42 ± 6.75 | 40.6 | 90 | 10 | 4(36.3) | 4(36.3) | 3(27.2) | 44.9 | 2.3 | 10.18 |
|  | VR-SPT（n=4） | 144 | 76.55 ± 4.37 | 41.66 | 50 | 50 | 0 | 4(100) | 0 | 40 | 3.25 | 12.5 |
|  | VR-MT（n=1） | 52 | 86.05 ± 5.6 | 23.07 | 100 | 0 | 0 | 1(100) | 0 | 38 | 1 | 8 |
|  | ACG（n=4） | 233 | 68.65 ± 4.39 | 37.76 | 100 | 0 | 0 | 0 | 4(100) | 63.75 | 3.5 | 10.25 |
| Executive function | VR-CCG（n=12） | 476 | 72.85 ± 3.8 | 47.68 | 91.6 | 8.4 | 1(8.4) | 5(41.6) | 6(50) | 35 | 3.3 | 8.3 |
|  | VR-CCG+SPT（n=9） | 359 | 77.11 ± 5.3 | 38.9 | 88.8 | 11.2 | 4(44.4) | 3(33.3) | 2(22.3) | 46.5 | 2.3 | 8.7 |
|  | VR-SPT（n=2） | 90 | 75.96 ± 6.14 | 34.4 | 100 | 0 | 0 | 2(100) | 0 | 50 | 3 | 18 |
|  | VR-SPT（n=2） | 167 | 68.91 ± 5.08 | 47.3 | 100 | 0 | 0 | 0 | 3(100) | 51.6 | 4 | 11 |
| Motor function | VR-CCG（n=1） | 68 | 72.6 ± 6.8 | 23.53 | 100 | 0 | 1(100) | 0 | 0 | 100 | 3 | 8 |
|  | VR-CCG+SPT（n=7） | 276 | 77.24 ± 6.32 | 34.42 | 85.71 | 14.29 | 1(14.4) | 4(57.1) | 2(28.5) | 48.57 | 2 | 11.7 |
|  | VR-SPT（n=4） | 171 | 77 ± 6.27 | 39.18 | 50 | 50 | 0 | 4(100) | 0 | 42.5 | 3 | 15.5 |

# Appendix 10 The strength of evidence

The evidence findings for all comparisons

(1) Global cognition function

Table S23 The strength of evidence for Global cognition function

 Major concerns Some concerns No concerns

| Comparison | Number of studies | Within  study bias | Reporting  bias | Indirectness | Imprecision | Heterogeneity | Incoherence | Confidence rating |
| --- | --- | --- | --- | --- | --- | --- | --- | --- |
| CG vs VR-CCG | 11 |  |  |  |  |  |  | Very low |
| CG vs VR-CCG+SPT | 6 |  |  |  |  |  |  | Very low |
| CG vs VR-SPT | 2 |  |  |  |  |  |  | Very low |
| CG vs ACG | 4 |  |  |  |  |  |  | Very low |
| VR-CCG vs ACG | 10 |  |  |  |  |  |  | Very low |
| VR-CCG+SPT vs ACG | 5 |  |  |  |  |  |  | Very low |

| Comparison | Number of studies | Within  study bias | Reporting  bias | Indirectness | Imprecision | Heterogeneity | Incoherence | Confidence rating |
| --- | --- | --- | --- | --- | --- | --- | --- | --- |
| VR-SPT vs ACG | 2 |  |  |  |  |  |  | Very low |
| VR-MT vs ACG | 1 |  |  |  |  |  |  | Very low |
| CG vs VR-MT | 0 |  |  |  |  |  |  | Very low |
| VR-CCG vs VR-CCG+SPT | 0 |  |  |  |  |  |  | Very low |
| VR-CCG vs VR-SPT | 0 |  |  |  |  |  |  | Very low |
| VR-CCG vs VR-MT | 0 |  |  |  |  |  |  | Very low |
| VR-CCG+SPT vs VR-SPT | 0 |  |  |  |  |  |  | Very low |
| VR-CCG+SPT vs VR-MT | 0 |  |  |  |  |  |  | Very low |
| VR-SPT vs VR-MT | 0 |  |  |  |  |  |  | Very low |

(2) Executive function

Table S24 The strength of evidence for executive function

Major concerns Some concerns No concerns

| Comparison | Number of studies | Within  study bias | Reporting  bias | Indirectness | Imprecision | Heterogeneity | Incoherence | Confidence rating |
| --- | --- | --- | --- | --- | --- | --- | --- | --- |
| CG vs VR-CCG | 5 |  |  |  |  |  |  | Very low |
| CG vs VR-CCG+SPT | 5 |  |  |  |  |  |  | Very low |
| CG vs VR-SPT | 1 |  |  |  |  |  |  | Very low |
| CG vs ACG | 3 |  |  |  |  |  |  | Very low |
| VR-CCG vs ACG | 7 |  |  |  |  |  |  | Very low |
| VR-CCG+SPT vs ACG | 4 |  |  |  |  |  |  | Very low |
| VR-SPT vs ACG | 1 |  |  |  |  |  |  | Very low |
| VR-CCG vs VR-CCG+SPT | 0 |  |  |  |  |  |  | Very low |
| VR-CCG vs VR-SPT | 0 |  |  |  |  |  |  | Very low |
| VR-CCG+SPT vs VR-SPT | 0 |  |  |  |  |  |  | Very low |

(3) Motor function

Table S25 The strength of evidence for motor function

Major concerns Some concerns No concerns

| Comparison | Number of studies | Within  study bias | Reporting  bias | Indirectness | Imprecision | Heterogeneity | Incoherence | Confidence rating |
| --- | --- | --- | --- | --- | --- | --- | --- | --- |
| CG vs VR-CCG | 1 |  |  |  |  |  |  | Very low |
| CG vs VR-CCG+SPT | 4 |  |  |  |  |  |  | Very low |
| CG vs VR-SPT | 3 |  |  |  |  |  |  | Very low |
| VR-CCG+SPT vs ACG | 3 |  |  |  |  |  |  | Very low |
| VR-SPT vs ACG | 1 |  |  |  |  |  |  | Very low |
| CG vs ACG | 0 |  |  |  |  |  |  | Very low |
| VR-CCG vs VR-CCG+SPT | 0 |  |  |  |  |  |  | Very low |
| VR-CCG vs VR-SPT | 0 |  |  |  |  |  |  | Very low |
| VR-CCG vs ACG | 0 |  |  |  |  |  |  | Very low |
| VR-CCG+SPT vs VR-SPT | 0 |  |  |  |  |  |  | Very low |
